# The relationship between living arrangements and higher use of hospital care at middle and older ages: to what extent do observed and unobserved individual characteristics explain this association?

**DOI:** 10.1186/s12889-019-7296-x

**Published:** 2019-07-29

**Authors:** Yaoyue Hu, Taina Leinonen, Karen van Hedel, Mikko Myrskylä, Pekka Martikainen

**Affiliations:** 1 Laboratory of Population Health, Max Planck Institute for Demographic Research, Konrad-Zuse-Straße 1, 18057 Rostock, Germany; 2 Population Research Unit, Department of Social Research, University of Helsinki, Helsinki, Finland; 3 Finnish Institute of Occupational Health, Helsinki, Finland; 4 Department of Social Policy, London School of Economics and Political Science, London, UK; 5 Centre for Health Equity Studies (CHESS), Stockholm University and Karolinska Institutet, Stockholm, Sweden

**Keywords:** Living arrangements, Hospital care use, Older population, Longitudinal registry data, Individual fixed-effects regression

## Abstract

**Background:**

Previous research has shown that certain living arrangements, such as living alone, are associated with worse health at older ages. We assessed the association between living arrangements and hospital care use among middle-aged and older adults, and investigated to what extent observed and unobserved individual characteristics explain this association.

**Methods:**

Longitudinal Finnish registry data for men and women aged 50–89 years were used for the period 1987–2007. The relationship between living arrangements (based on whether an individual lived with a partner, other adults or alone, and whether they lived with minor/adult children) and heavy hospital care use (i.e., having been in hospital for 8 or more days in a year) was studied. First, we applied logistic regression models and linear probability models controlling for observed time-invariant factors (socioeconomic status measured by education, labour force status, and household income; and marital status), and then individual linear probability models with fixed-effects to further account for unobserved time-invariant individual characteristics in the measurement period. Analyses were done separately for 10 year age-groups.

**Results:**

In the logistic regression models, men and women who lived alone had higher crude odds of heavy hospital care use than those living only with their partner. These odds ratios were highest for men and women in the youngest age category (50–59 years, 1.72 and 1.36 respectively) and decreased with age. Adjusting for observed time-invariant socioeconomic status attenuated these odds by 14–40%, but adjusting for marital status did not affect the results. Lower odds were observed among adults aged 50–59 years who lived with their partner and (minor or adult) children. But odds were higher for individuals aged 60–79 years who co-resided with their adult children, regardless of whether they lived with a partner. Adjusting for observed time-invariant factors generally did not change these results. After further adjusting for unobserved time-invariant individual characteristics in the individual fixed-effects models, most of these associations largely attenuated or disappeared, particularly for ages 80–89 years.

**Conclusions:**

The association between living arrangements and higher use of hospital care at middle and older ages is largely explained by socioeconomic disadvantage and unobserved time-invariant individual characteristics.

**Electronic supplementary material:**

The online version of this article (10.1186/s12889-019-7296-x) contains supplementary material, which is available to authorized users.

## Background

Developed countries have undergone a continuous expansion of single-person households among older adults, presenting profound implications on individual health and functioning and on demands for both informal and formal care [1–3]. Detrimental effects of living alone have been shown on physical health, mental health, institutionalisation, and survival [4–10]. However, living alone has also been demonstrated not to be a good predictor of hospital admission or length of hospital stay [11–14], nor to be associated with self-rated health or physical limitations among Europeans aged 65–84 years [15] or with mortality among Americans aged 70 years and older [16]. Conflicting evidence from a longitudinal study of American women aged 60–72 years reported protective effects of living alone on functional declines, mental health, and vitality [17].

Co-residence with children in relation to older parents’ health is less studied [15]. Co-residence with (adult) children was found to be associated with worsened psychological well-being of mothers at late-middle and early-old ages [18] and with heightened risk of having two or more health conditions among lone mothers aged 65 years and over [19]; these associations were not observed among men. In a study of men and women aged 51–61 years, no differences in self-rated health, mobility limitation, or depressive symptoms were detected between married couples living with and without children, but worse health was found for lone mothers living with children [5].

The relationship between living arrangements and health is likely to be gendered and age-specific [19–22]. Compared to men, women are more likely to feel strong family obligations, keep family bonds, and be involved in assistance and caregiving [23], which bring both greater burdens and greater benefits from family relationships [5, 23, 24]. Firm evidence has been found among young adults that living with a partner is beneficial for health due to a variety of material and psychosocial reasons [25]. For the elderly, similar benefits may exist, but their good health may enable them to live independently rather than vice versa [16, 26]. The association between living alone and health thus may decrease with age. Furthermore, Lawton and colleagues [27] asserted that any observed association between living arrangements and health may be related to factors that are idiosyncratic to the individual. To address this, individual fixed-effects regression – a technique controlling for all observed and unobserved time-invariant individual characteristics [28, 29] – could be useful. We are aware of only one recent study applying this technique to investigate the effect of returning-home adult children on older parents’ quality of life [30].

By exploiting longitudinal Finnish registry data, we investigated the relationship between living arrangements and hospital care use among middle-aged and older adults aged 50–89 years, and how this relationship differed by gender, age, and when controlling for observed and unobserved time-invariant individual characteristics. We focused on hospital care use to capture middle-aged and older adults’ ill health given that illness represents the most immediate causes of hospitalisation [31, 32].

## Methods

### Study design

The data came from an 11% random sample of the population permanently residing in Finland at the end of any of the years between 1987 and 2006, obtained from the longitudinal population data file of Statistics Finland. The register-based information was based on routinely collected administrative data updated at the end of each year in 1987–2006. Individual-level linkages were performed with the help of an unique identifier available for all persons. For each person, hospital care use was extracted from the national Hospital Discharge Register. In this study we included individuals aged 50–89 years at the end of any of the years 1987–2006, and followed their hospital care use in each following calendar year (1988–2007). We did not include individuals aged 90 years and beyond as they were increasingly likely to be in institutional care, from where they could to some extent receive medical care. Hospital care use of the population aged 90 years and over may therefore be an underestimation of all care needs in our data. Individuals were excluded if they were not part of the dwelling population at the end of either 1985 or 1987, or did not reside in Finland in any given year during our study period. The sample was stratified by their current age (50–59, 60–69, 70–79, and 80–89 years). The maximum measurement period for a study subject in one age group was ten years, and a study subject could appear in more than one age group.

### Living arrangements

At the end of each year in 1987–2006, information on family type (e.g., married/cohabiting couple with or without children, lone parents), own status in a family (parent or adult child), number of minor children (< 18 years old), and number of persons living in the household-dwelling unit was updated. Persons living in the household-dwelling unit are the permanent occupants of a dwelling. Those who live in an institutionalised setting or in residential homes, live abroad, are homeless, or are registered as unknown are not part of the dwelling population.

Using this information, living arrangements were categorised into: 1) living only with a partner (spouse or cohabiting partner); 2) living with a partner and at least one minor child (and possibly adult children); 3) living with a partner and only adult children; 4) being a lone parent living with at least one minor child; 5) being a lone parent living with only adult children; 6) living alone (living in a single-person household-dwelling unit); 7) living with other persons than nuclear family members (living in a household-dwelling unit with several persons who are not the partner or the children); 8) other living arrangements (e.g., living in residential homes or institutions). Since long-term care is universally accessible in Finland and can be provided through home-based or institutional care, the community-living older persons could also be receiving home-based long-term care. We excluded study subjects aged 70–89 years living with at least one minor child due to the small proportion of this living arrangement (< 0.1%).

### Hospital care use

Hospital discharge records between 1 January 1988 and 31 December 2007 were extracted. Multiple hospitalisation episodes could occur in a given year. Hospital episodes spanning over two calendar years were split into two; one for each calendar year. Annual total number of days spent in hospital for each calendar year was calculated. Ill health was captured by dichotomising the hospital days into 0–7 (coded as 0) vs. 8 or more days (i.e., heavy hospital care use coded as 1) based on the population median.

### Covariates

Current age and region of residence (south, north, east, and west) were time-varying and measured annually. Socioeconomic status (SES) and marital status were time-invariant and assessed when study subjects entered into the 10-year age groups. SES was reflected by educational attainment (compulsory education only, upper secondary school, and tertiary education), labour force status (employed, unemployed, pensioners, and other), and household taxable income divided by the number of consumption units in the household using the Organisation for Economic Co-operation and Development equivalence scale [33]. Household income was categorised into tertiles by gender, 5-year age group, and year separately. Marital status (unmarried, married, divorced, and widowed) was used to distinguish reasons for living alone at baseline.

### Statistical analyses

The relationship between living arrangements and heavy hospital use was first analysed using logistic regression. Logistic regression uses all observed information available (i.e., both between- and within-individual variations), and we estimated clustered robust standard errors to account for the correlated observations from the same individual. In contrast, the individual fixed-effects (FE) regression only uses within-individual changes over time where study subjects serve as their own controls (i.e., between-individual variation is not used in the estimation), and thus controls for all observed and unobserved time-invariant individual characteristics in the measurement period [28, 29]. By comparing the results from the ordinary regression model controlling for observed covariates at baseline (i.e., observed time-invariant individual characteristics) with those from the individual FE model, the role of unobserved time-invariant individual characteristics in the relationship between living arrangements and hospital care use could be assessed.

The individual FE logistic regression nevertheless does not include study subjects in its analytic sample whose outcome remain unchanged over time (i.e., always coded as 0 or as 1) [29]. This discrepancy of the analytic sample between logistic regression and individual FE logistic regression hinders direct comparison of results from the two different models to assess the confounding caused by unobserved time-invariant individual characteristics. As a result, we employed the linear probability model (LPM) – a widely used alternative to logistic regression for analysing binary outcome with ordinary least squares (OLS) – and the individual linear probability model with fixed-effects (LPM-FE) to compare them with [34, 35]. The interpretation of coefficients from LPM is simple which indicates how much of the probability of heavy hospital care use increases or decreases for study subjects with certain type of living arrangements compared to those living with a partner only (reference group). This interpretation also implies that coefficients from LPM are on an absolute scale, i.e. not on a relative scale as those from logistic regression models are. To keep the scale of results consistent, we transformed the coefficients from LPM and LPM-FE into relative difference via dividing the predicted probability of heavy hospital care use for one living arrangement by that for the reference living arrangement. The 95% confidence intervals of relative difference were calculated using the delta method [36].

For the logistic regression, three models were estimated adjusting for time-varying current age dummies and region of residence (Model 1), additionally for time-invariant SES (Model 2), and further for time-invariant marital status (Model 3). We further estimated LPM controlling for all covariates in Model 3 and individual LPM-FE adjusting for time-varying current age dummies and region of residence. For sensitivity analyses, the estimations were repeated using the average number of days spent in hospital per hospital episode in a given year (i.e., the total hospital days in a year divided by the total number of hospitalisation episodes) as the outcome, and categorised as 0–4 vs. 5 or more days per episode using the population median. All analyses were performed separately for men and women and for the four age groups using Stata 15 (StataCorp, 2017). Because the four 10-year age groups were based on time-varying age, the LPM-FE accounts for all individual characteristics that were time invariant in the 10-year observation window.

## Results

The proportion of both men and women living with partner and/or children decreased with age, whereas the proportion of living alone increased with age particularly among women (Table 1). More than 70% of men and women experienced at least one change of their living arrangement in the 10-year observation window (50–59 years: 72%, 60–69 years: 78%, 70–79 years: 79%, 80–89 years: 74%). In general, for both genders and all age groups, the proportion of heavy hospital care use was higher among those living alone or living with other persons than among those with other living arrangements. Compared to younger study subjects, older ones tended to have less education, be retired, and be widowed (see Additional file 1).

**Table 1:** Distribution of living arrangements and heavy hospital care use by living arrangements, gender, and age groups

	Men		Women	
	%	8+ hospital days in a year (%)^§^	%	8+ hospital days in a year (%)^§^
50–59 years				
Total observations	683,709		702,204	
Living with a partner only	39.3	4.8	43.4	4.3
Living with a partner & 1+ minor child	16.7	3.5	9.1	3.4
Living with a partner & adult children	19.5	4.3	18.2	4.0
Lone parent living with 1+ minor child	0.6	4.6	2.0	4.1
Lone parent living with adult children	1.4	5.7	4.5	5.0
Living alone	15.8	7.8	19.6	5.7
Living with others	4.0	8.6	2.4	7.1
Other	2.8	8.0	1.0	10.4
60–69 years				
Total observations	479,938		569,573	
Living with a partner only	62.1	9.1	50.2	7.0
Living with a partner & 1+ minor child	2.1	7.8	0.2	4.5
Living with a partner & adult children	12.9	9.3	8.5	7.5
Lone parent living with 1+ minor child	0.1	10.8	< 0.1	6.7
Lone parent living with adult children	1.2	11.3	4.9	8.8
Living alone	16.9	13.0	31.6	8.8
Living with others	3.9	12.4	3.9	10.1
Other	0.9	21.8	0.6	25.6
70–79 years				
Total observations	282,142		457,226	
Living with a partner only	65.2	18.5	32.4	15.0
Living with a partner & adult children	8.0	20.2	3.4	16.3
Lone parent living with adult children	1.7	22.0	6.3	18.5
Living alone	20.4	22.4	49.3	17.8
Living with others	3.6	22.0	6.7	20.6
Other	1.2	44.5	1.9	43.0
80–89 years				
Total observations	89,798		216,814	
Living with a partner only	50.9	31.9	12.3	28.4
Living with a partner & adult children	5.2	31.3	1.0	27.2
Lone parent living with adult children	2.9	35.3	7.0	30.9
Living alone	31.0	34.4	60.7	31.5
Living with others	5.7	38.1	11.3	35.6
Other	4.4	46.4	7.9	43.9

Note: Having at least one minor child in the household was rare for ages 70 years over, therefore its effect was not estimated for ages 70 years and over

^§^: percentage of heavy hospital care use within each living arrangement category

**Fig. 1:**
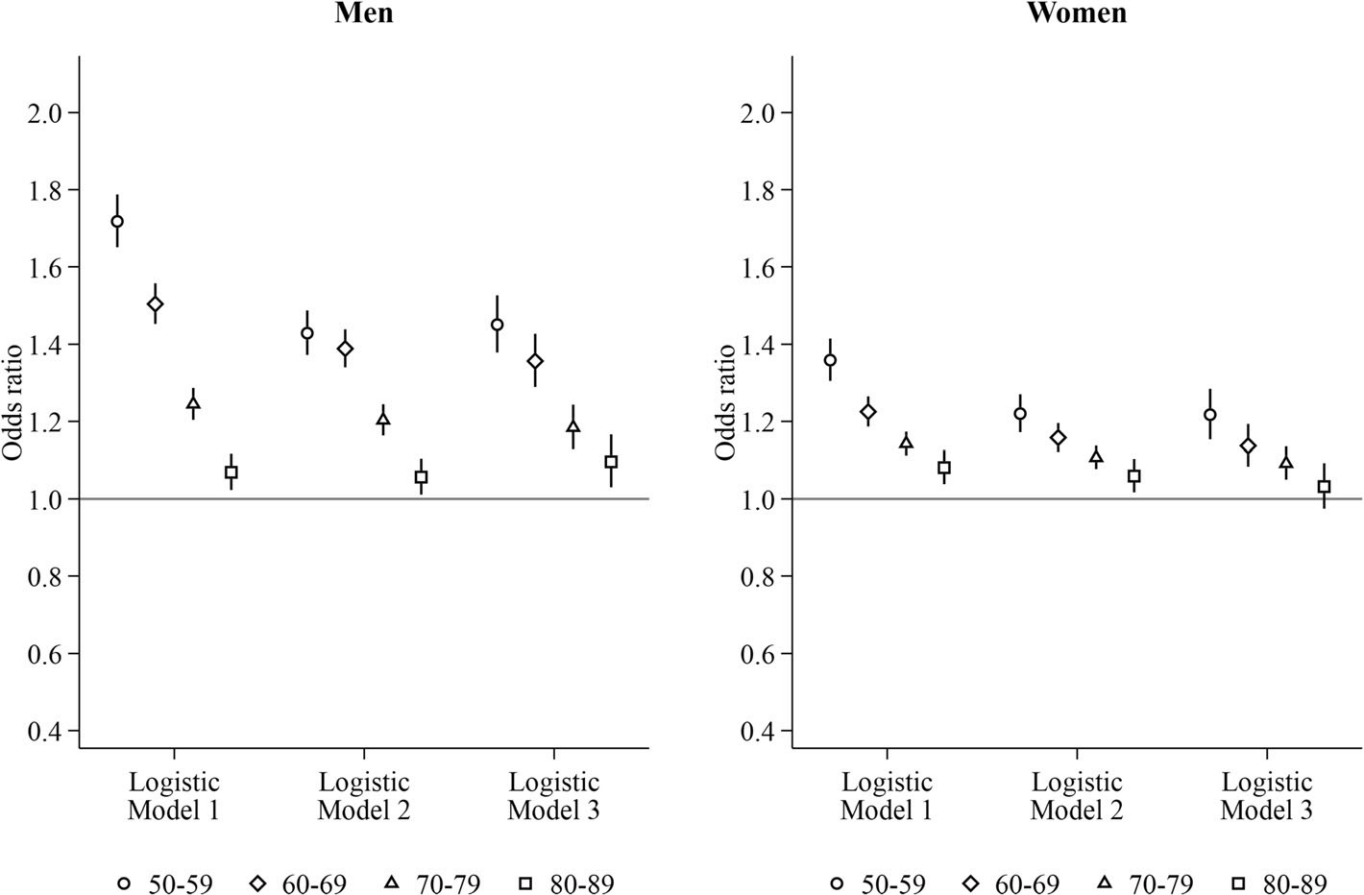
Risk of heavy hospital care use for men and women living alone. Reference group: living with the partner only. Logistic Model 1: adjusting for time-varying current age dummies and region of residence. Logistic Model 2: Model 1 + education, household income, and labour force status measured at the time when study subjects entered into the current age group. Logistic Model 3: Model 2 + marital status measured at the time when study subjects entered into the current age group

After adjusting for time-varying current age dummies and region of residence using logistic regression (Fig. 1, Model 1; numerical values see Tables 2-3), 72 and 36% higher odds of heavy hospital care use were found among men and women aged 50–59 years living alone (men: odds ratio [OR]: 1.72, 95% confidence interval [CI]: 1.65–1.79; women: 1.36, 95% CI: 1.31–1.41) than those living only with their partner, respectively. The heightened odds gradually decreased with age for both men (60–69 years: 1.50, 95% CI: 1.45–1.56; 70–79 years: 1.24, 95% CI: 1.20–1.29; 80–89 years: 1.07, 95% CI: 1.02–1.12) and women (60–69 years: 1.23, 95% CI: 1.19–1.26; 70–79 years: 1.14, 95% CI: 1.11–1.17; 80–89 years: 1.08, 95% CI: 1.04–1.13). These associations attenuated by 14–40% when controlling for time-invariant SES (Model 2). Additional adjustment for time-invariant marital status (Model 3) only very slightly changed these associations. The highly similar results from logistic Model 3 and LPM (relative difference) validated the rationale to use LPM (see model coefficients and predicted probabilities on the absolute scale from LPM and LPM-FE in Additional files 2-3). In the individual LPM-FE model, compared to the LPM, the effect of living alone attenuated by 69% for men aged 50–69 years and by 38% for women aged 50–59 years. Living alone was no longer associated with heavy hospital care use for either men or women aged 80–89 years in the LPM-FE model.

**Table 2:** Living arrangements and risk of being hospitalised for 8 or more days in a year among men, by 10-year age groups

	Logistic Model 1	Logistic Model 2	Logistic Model 3	LPM	LPM-FE
	OR (95% CI)	OR (95% CI)	OR (95% CI)	Relative difference (95% CI)	Relative difference (95% CI)
50–59 years					
Living with a partner only	Ref	Ref	Ref	Ref	Ref
Living with a partner & 1+ minor child	0.78 (0.75, 0.82)	0.82 (0.78, 0.86)	0.81 (0.78, 0.86)	0.86 (0.82, 0.89)	1.09 (1.03, 1.16)
Living with a partner & adult children	0.91 (0.87, 0.94)	0.94 (0.90, 0.98)	0.94 (0.90, 0.98)	0.95 (0.92, 0.99)	1.06 (1.02, 1.10)
Lone parent living with 1+ minor child	1.03 (0.87, 1.22)	1.01 (0.85, 1.20)	0.96 (0.81, 1.14)	0.94 (0.79, 1.09)	1.26 (1.06, 1.46)
Lone parent living with adult children	1.24 (1.11, 1.39)	1.20 (1.07, 1.34)	1.13 (1.00, 1.26)	1.11 (0.98, 1.23)	1.20 (1.06, 1.33)
Living alone	1.72 (1.65, 1.79)	1.43 (1.37, 1.49)	1.45 (1.38, 1.53)	1.45 (1.38, 1.52)	1.14 (1.07, 1.22)
Living with others	1.85 (1.74, 1.98)	1.41 (1.32, 1.51)	1.48 (1.37, 1.59)	1.51 (1.40, 1.61)	0.99 (0.89, 1.10)
Other	1.79 (1.65, 1.95)	1.34 (1.23, 1.46)	1.46 (1.33, 1.61)	1.47 (1.33, 1.60)	0.81 (0.68, 0.94)
60–69 years					
Living with a partner only	Ref	Ref	Ref	Ref	Ref
Living with a partner & 1+ minor child	1.00 (0.90, 1.10)	1.06 (0.96, 1.17)	1.06 (0.96, 1.17)	1.05 (0.98, 1.13)	1.22 (1.11, 1.32)
Living with a partner & adult children	1.07 (1.03, 1.11)	1.08 (1.03, 1.12)	1.08 (1.03, 1.12)	1.07 (1.03, 1.11)	1.06 (1.01, 1.10)
Lone parent living with 1+ minor child	1.35 (0.90, 2.02)	1.36 (0.90, 2.06)	1.28 (0.84, 1.94)	1.22 (0.79, 1.64)	1.51 (1.11, 1.91)
Lone parent living with adult children	1.27 (1.13, 1.43)	1.24 (1.10, 1.39)	1.19 (1.05, 1.34)	1.16 (1.03, 1.29)	1.02 (0.88, 1.16)
Living alone	1.50 (1.45, 1.56)	1.39 (1.34, 1.44)	1.36 (1.29, 1.43)	1.32 (1.26, 1.38)	1.10 (1.04, 1.17)
Living with others	1.42 (1.33, 1.51)	1.31 (1.23, 1.39)	1.30 (1.21, 1.41)	1.28 (1.19, 1.36)	1.06 (0.96, 1.16)
Other	2.84 (2.53, 3.18)	2.53 (2.26, 2.83)	2.53 (2.24, 2.85)	2.27 (2.05, 2.48)	0.80 (0.66, 0.94)
70–79 years					
Living with a partner only	Ref	Ref	Ref	Ref	Ref
Living with a partner & adult children	1.11 (1.05, 1.17)	1.10 (1.05, 1.16)	1.10 (1.05, 1.16)	1.08 (1.04, 1.12)	1.01 (0.95, 1.07)
Lone parent living with adult children	1.17 (1.06, 1.29)	1.16 (1.06, 1.28)	1.13 (1.02, 1.26)	1.06 (0.96, 1.16)	1.11 (0.99, 1.23)
Living alone	1.24 (1.20, 1.29)	1.20 (1.16, 1.24)	1.18 (1.13, 1.24)	1.11 (1.05, 1.16)	1.09 (1.04, 1.14)
Living with others	1.19 (1.11, 1.28)	1.15 (1.07, 1.23)	1.16 (1.06, 1.26)	1.09 (1.01, 1.17)	1.03 (0.95, 1.12)
Other	3.17 (2.87, 3.49)	3.04 (2.75, 3.36)	3.01 (2.72, 3.34)	2.21 (2.07, 2.35)	0.57 (0.49, 0.65)
80–89 years					
Living with a partner only	Ref	Ref	Ref	Ref	Ref
Living with a partner & adult children	0.94 (0.85, 1.03)	0.93 (0.85, 1.02)	0.93 (0.85, 1.02)	0.95 (0.89, 1.01)	0.93 (0.83, 1.04)
Lone parent living with adult children	1.07 (0.95, 1.20)	1.06 (0.94, 1.20)	1.08 (0.95, 1.22)	1.10 (0.99, 1.20)	1.07 (0.94, 1.20)
Living alone	1.07 (1.02, 1.12)	1.06 (1.01, 1.10)	1.10 (1.03, 1.17)	1.11 (1.04, 1.17)	1.02 (0.97, 1.06)
Living with others	1.20 (1.11, 1.31)	1.19 (1.09, 1.29)	1.27 (1.15, 1.39)	1.21 (1.12, 1.29)	1.03 (0.96, 1.11)
Other	1.56 (1.42, 1.71)	1.53 (1.39, 1.68)	1.57 (1.42, 1.74)	1.37 (1.28, 1.46)	0.53 (0.48, 0.59)

LPM: linear probability model, adjusting for all covariates in Model 3

LPM-FE: linear probability model with fixed-effects adjusting for current age dummies and region of residence

OR: odds ratio; CI: confidence interval; Ref: reference category

Logistic Model 1: adjusting for current age dummies and region of residence;

Logistic Model 2: Model 1 + education, household income, and labour force status at time of entry to the age group;

Logistic Model 3: Model 2 + marital status at time of entry to the age group

**Table 3:** Living arrangements and risk of being hospitalised for 8 or more days in a year among women, by 10-year age groups

	Logistic Model 1	Logistic Model 2	Logistic Model 3	LPM	LPM-FE
	OR (95% CI)	OR (95% CI)	OR (95% CI)	Relative difference (95% CI)	Relative difference (95% CI)
50–59 years					
Living with a partner only	Ref	Ref	Ref	Ref	Ref
Living with a partner & 1+ minor child	0.83 (0.78, 0.88)	0.88 (0.83, 0.94)	0.89 (0.84, 0.94)	0.91 (0.86, 0.96)	1.09 (1.01, 1.16)
Living with a partner & adult children	0.95 (0.91, 0.99)	0.97 (0.93, 1.02)	0.98 (0.94, 1.02)	0.98 (0.95, 1.02)	1.07 (1.02, 1.11)
Lone parent living with 1+ minor child	1.00 (0.90, 1.11)	0.94 (0.85, 1.05)	0.94 (0.84, 1.05)	0.93 (0.83, 1.03)	1.16 (1.02, 1.30)
Lone parent living with adult children	1.19 (1.11, 1.27)	1.07 (1.00, 1.14)	1.06 (0.99, 1.15)	1.06 (0.98, 1.14)	1.14 (1.05, 1.24)
Living alone	1.36 (1.31, 1.41)	1.22 (1.17, 1.27)	1.22 (1.15, 1.28)	1.21 (1.15, 1.27)	1.13 (1.05, 1.20)
Living with others	1.69 (1.55, 1.85)	1.24 (1.14, 1.36)	1.26 (1.15, 1.39)	1.29 (1.16, 1.42)	1.03 (0.90, 1.16)
Other	2.61 (2.28, 2.97)	1.67 (1.47, 1.90)	1.74 (1.51, 2.00)	1.88 (1.61, 2.15)	0.83 (0.61, 1.04)
60–69 years					
Living with a partner only	Ref	Ref	Ref	Ref	Ref
Living with a partner & 1+ minor child	0.77 (0.53, 1.11)	0.77 (0.53, 1.12)	0.77 (0.54, 1.12)	0.83 (0.62, 1.04)	1.13 (0.88, 1.38)
Living with a partner & adult children	1.09 (1.03, 1.14)	1.09 (1.03, 1.14)	1.09 (1.03, 1.15)	1.08 (1.03, 1.12)	1.03 (0.97, 1.09)
Lone parent living with 1+ minor child	1.16 (0.66, 2.03)	1.01 (0.57, 1.77)	0.99 (0.56, 1.74)	0.97 (0.50, 1.45)	1.20 (0.71, 1.70)
Lone parent living with adult children	1.23 (1.15, 1.30)	1.15 (1.08, 1.22)	1.12 (1.05, 1.20)	1.11 (1.03, 1.18)	1.08 (0.99, 1.17)
Living alone	1.23 (1.19, 1.26)	1.16 (1.12, 1.20)	1.14 (1.08, 1.19)	1.12 (1.07, 1.18)	1.11 (1.05, 1.17)
Living with others	1.40 (1.31, 1.49)	1.30 (1.22, 1.38)	1.28 (1.19, 1.38)	1.27 (1.18, 1.36)	1.05 (0.95, 1.14)
Other	4.35 (3.79, 4.98)	3.79 (3.30, 4.34)	3.76 (3.26, 4.34)	3.31 (2.95, 3.66)	0.55 (0.38, 0.73)
70–79 years					
Living with a partner only	Ref	Ref	Ref	Ref	Ref
Living with a partner & adult children	1.09 (1.02, 1.16)	1.09 (1.02, 1.16)	1.09 (1.02, 1.16)	1.07 (1.01, 1.12)	0.96 (0.89, 1.03)
Lone parent living with adult children	1.16 (1.11, 1.22)	1.14 (1.09, 1.20)	1.12 (1.06, 1.19)	1.10 (1.02, 1.17)	1.07 (1.01, 1.14)
Living alone	1.14 (1.11, 1.17)	1.11 (1.08, 1.14)	1.09 (1.05, 1.14)	1.07 (1.01, 1.14)	1.04 (1.01, 1.07)
Living with others	1.32 (1.26, 1.38)	1.30 (1.24, 1.36)	1.29 (1.22, 1.36)	1.23 (1.15, 1.31)	0.98 (0.93, 1.04)
Other	3.42 (3.19, 3.67)	3.29 (3.07, 3.53)	3.26 (3.03, 3.52)	2.48 (2.32, 2.63)	0.46 (0.40, 0.52)
80–89 years					
Living with partner only	Ref	Ref	Ref	Ref	Ref
Living with partner & adult children	0.92 (0.80, 1.05)	0.92 (0.80, 1.06)	0.92 (0.80, 1.06)	0.95 (0.86, 1.04)	0.98 (0.86, 1.10)
Lone parent living with adult children	1.02 (0.96, 1.08)	1.01 (0.95, 1.08)	0.98 (0.91, 1.06)	0.97 (0.90, 1.04)	0.99 (0.92, 1.06)
Living alone	1.08 (1.04, 1.13)	1.06 (1.02, 1.10)	1.03 (0.97, 1.09)	1.01 (0.94, 1.07)	1.03 (0.99, 1.07)
Living with others	1.23 (1.17, 1.30)	1.23 (1.17, 1.30)	1.21 (1.13, 1.29)	1.12 (1.04, 1.19)	0.98 (0.93, 1.03)
Other	1.55 (1.46, 1.65)	1.51 (1.42, 1.61)	1.48 (1.38, 1.59)	1.28 (1.19, 1.36)	0.44 (0.41, 0.49)

LPM: linear probability model, adjusting for all covariates in Model 3

LPM-FE: linear probability model with fixed-effects adjusting for current age dummies and region of residence

OR: odds ratio; CI: confidence interval; Ref: reference category

Logistic Model 1: adjusting for current age dummies and region of residence;

Logistic Model 2: Model 1 + education, household income, and labour force status at time of entry to the age group;

Logistic Model 3: Model 2 + marital status at time of entry to the age group

**Fig. 2:**
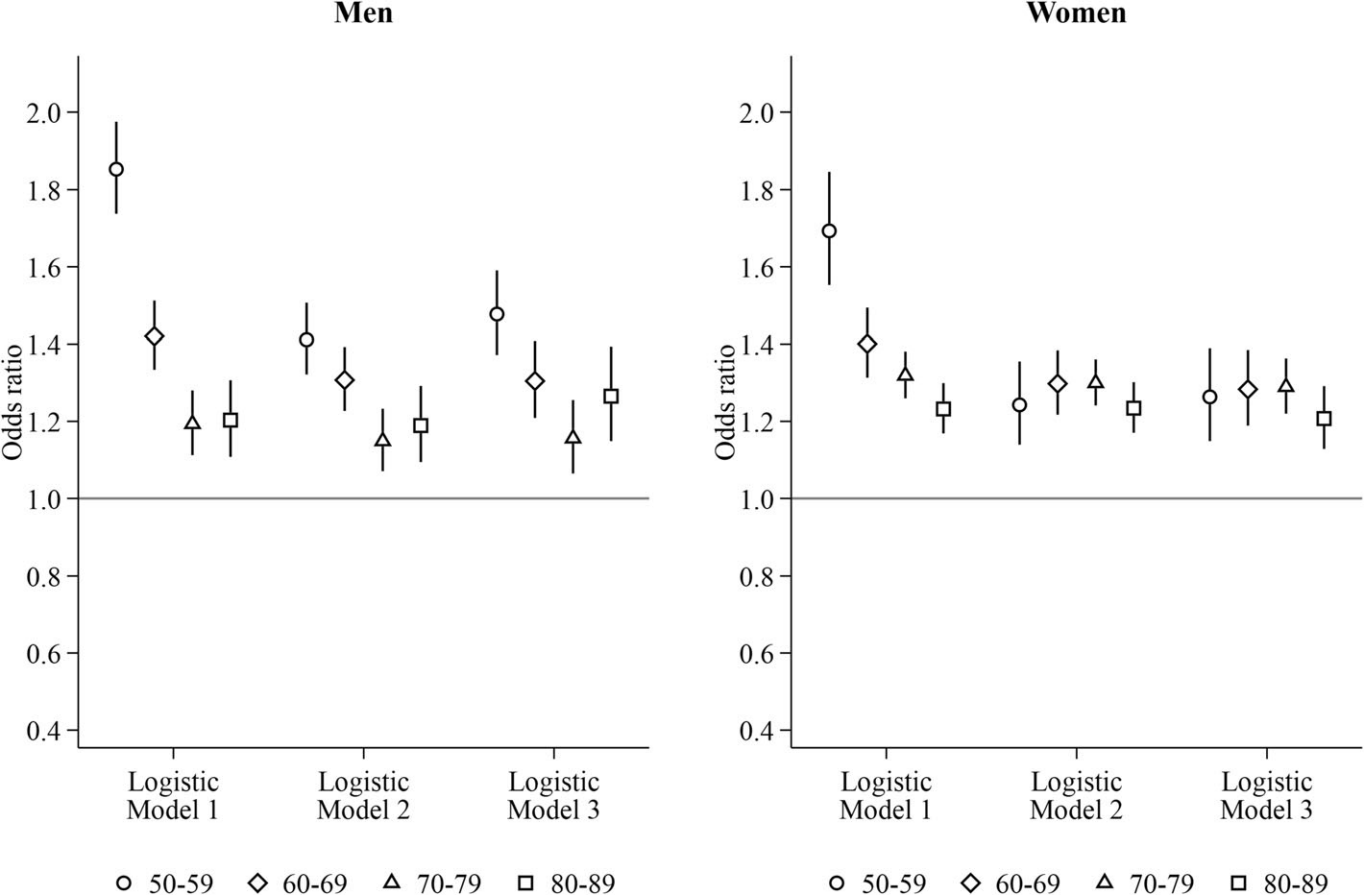
Risk of heavy hospital care use for men and women living with other persons. Reference group: living with the partner only. Logistic Model 1: adjusting for time-varying current age dummies and region of residence. Logistic Model 2: Model 1 + education, household income, and labour force status measured at the time when study subjects entered into the current age group. Logistic Model 3: Model 2 + marital status measured at the time when study subjects entered into the current age group

A similar age pattern was found for living with other persons (Fig. 2, Model 1). The elevated odds of heavy hospital care use associated with living with other persons nevertheless became similar across all age groups among women after adjustment for time-invariant SES (50–59 years: 1.24, 95% CI: 1.14–1.36; 60–69 years: 1.30, 95% CI: 1.22–1.38; 70–79 years: 1.30, 95% CI: 1.24–1.36; 80–89 years: 1.23, 95% CI: 1.17–1.30). In the individual LPM-FE model, no association was found.

**Fig. 3:**
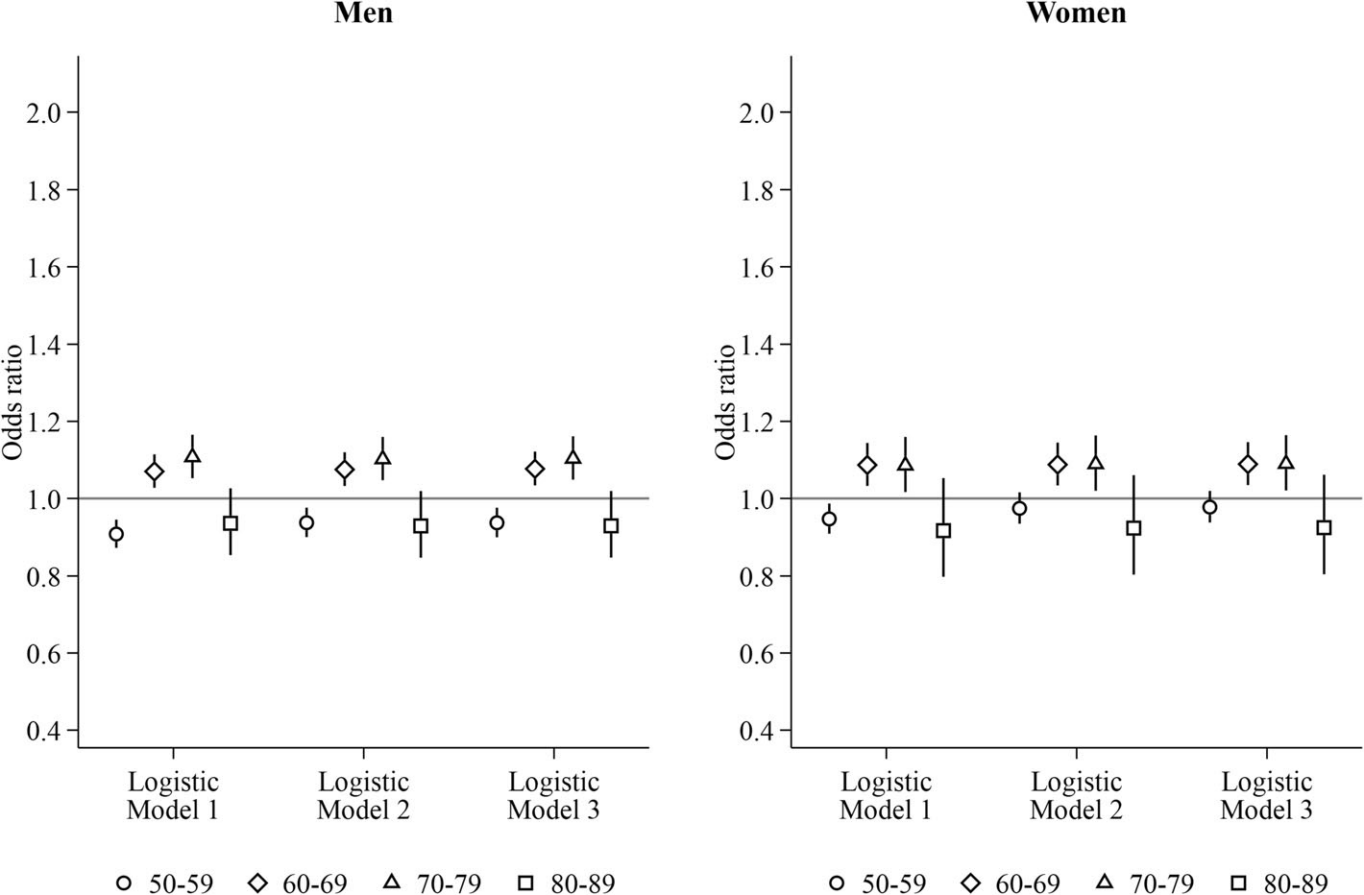
Risk of heavy hospital care use for men and women living with partner and adult children. Reference group: living with the partner only. Logistic Model 1: adjusting for time-varying current age dummies and region of residence. Logistic Model 2: Model 1 + education, household income, and labour force status measured at the time when study subjects entered into the current age group. Logistic Model 3: Model 2 + marital status measured at the time when study subjects entered into the current age group

**Fig. 4:**
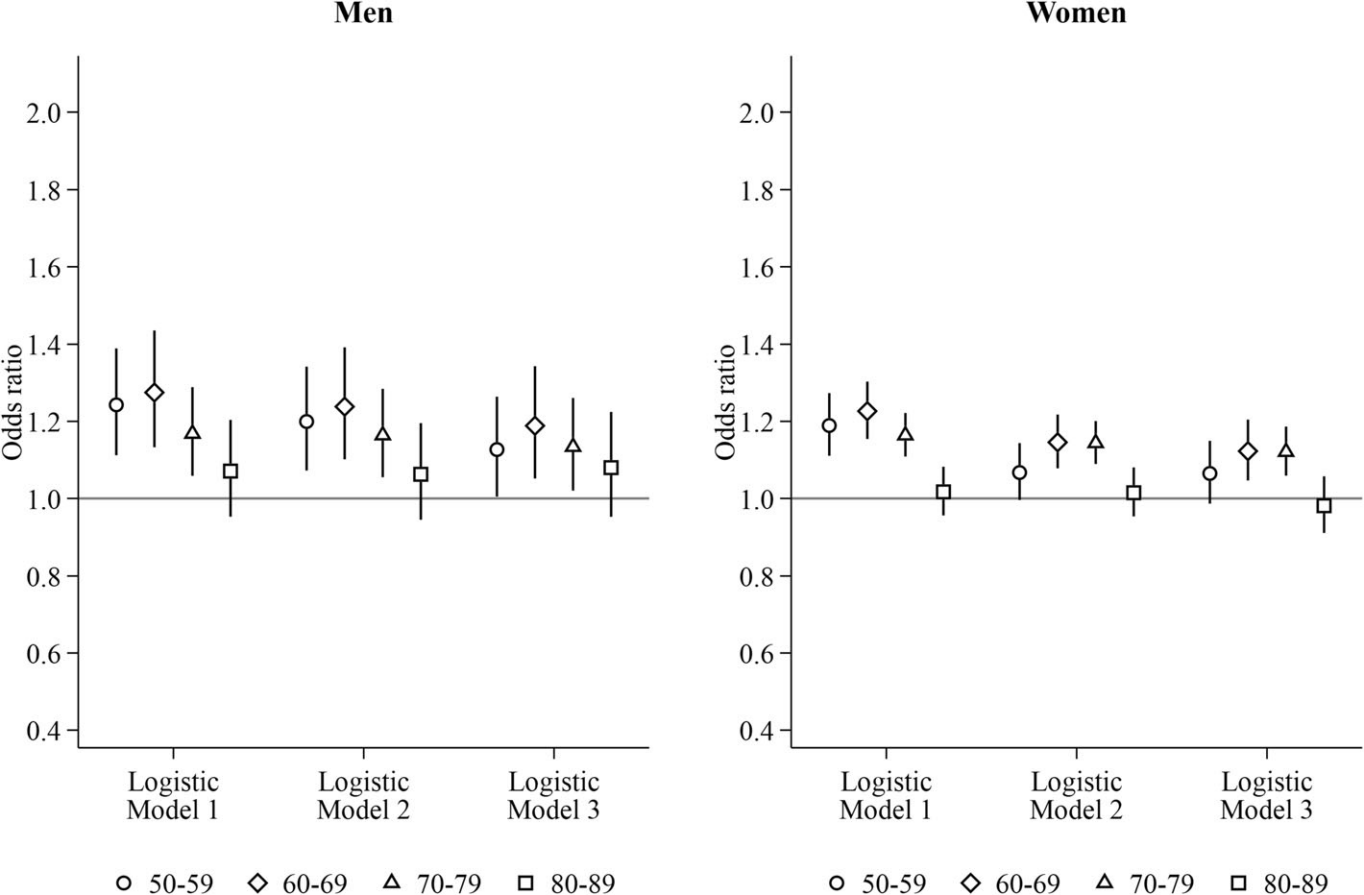
Risk of heavy hospital care use for lone parents living with adult children. Reference group: living with the partner only. Logistic Model 1: adjusting for time-varying current age dummies and region of residence. Logistic Model 2: Model 1 + education, household income, and labour force status measured at the time when study subjects entered into the current age group. Logistic Model 3: Model 2 + marital status measured at the time when study subjects entered into the current age group

Regardless of the presence of partner in the household, men and women co-residing with adult children had higher odds of heavy hospital care use than those living only with their partner for ages 60–79 years using logistic regression (Figs. 3-4). For ages 50–59 years, men and women living with their partner and adult children had lower odds of heavy hospital care use, whereas lone parents living with adult children had higher odds. In the LPM-FE models, living with a partner and adult children was associated with elevated probability of heavy hospital care use among men aged 50–69 years and among women aged 50–59 years. Heightened probability in relation to being a lone parent living with adult children were found for both genders aged 50–59 years (relative difference in men: 1.20, 95% CI: 1.06–1.33; relative different in women: 1.14, 95% CI: 1.05–1.24) and among women aged 70–79 years (1.07, 95% CI: 1.01–1.14) in the LPM-FE models.

Men and women aged 50–59 years living with their partner and at least one minor child had lower odds of heavy hospital use than their counterparts living only with their partner using logistic regression (Model 3, men: 0.81, 95% CI: 0.78–0.86; women: 0.89, 95% CI: 0.84–0.94), but no association was found for lone parents aged 50–69 years living with at least one minor child (Additional files 4-5). In the LPM-FE models, among men, living with at least one minor child, regardless of the present of partner, was associated with higher probability of heavy hospital care use (relative difference in lone fathers aged 50–59 years: 1.26, 95% CI: 1.06–1.46; lone fathers aged 60–69 years: 1.51, 95% CI: 1.11–1.91; men aged 50–59 years also living with a partner: 1.09, 95% CI: 1.03–1.16; men aged 60–69 years also living with a partner: 1.22, 95% CI: 1.11–1.32). Similarly increased probability of heavy hospital care use was also found among women aged 50–59 years living with minor children.

We repeated our analyses using the average number of days spent in hospital per hospital episode in a year (0–4 vs. 5 or more days using the population median). The pattern of results remained (Additional files 6-7), but in the LPM-FE models, the effect of living alone was more evident among men aged 60–79 years when using average number of days spent in hospital per hospital episode in a year as the outcome (relative difference for age 60–69 years: 1.15, 95% CI: 1.08–1.21; 70–79 years: 1.12, 95% CI: 1.07–1.17).

## Discussion

We found 72 and 36% higher odds of heavy hospital care use among men and women aged 50–59 years living alone than among those living only with a partner, respectively. These elevated odds decreased with age for both genders. A similar age pattern was observed for living with other persons. Men and women aged 60–79 years co-residing with adult children had elevated odds of heavy hospital care use; this effect largely disappeared in the individual fixed-effects models. The protective effect of co-residing with at least one minor children found among men and women aged 50–59 years who were also living with their partner in the logistic regression models and linear probability models reversed in the individual linear probability fixed-effects models.

### Living alone

Compared to living alone, living with a partner may have health benefits because the partner may bring more economic resources into the household, provide better monitoring of health and social control of behaviours, encourage help-seeking behaviours at early stage of illness, and contribute to more social ties and social support [5, 22, 24, 26, 37, 38]. These explanations are partly supported by our findings that adjusting for various SES indicators significantly attenuated the effect of living alone on hospital care use. The decreasing effect of living alone with age we found may support the health selection hypothesis – only healthy and well-functioning elderly can continue to live independently in the community [15, 16, 26]. However the alternative explanation that the elderly may become universally frail at old-old ages (≥70 years), a stage of life when biological mechanisms override social mechanisms – such as living arrangements – on health, cannot be ruled out [39].

When all observed and unobserved time-invariant individual characteristics in the measurement period were controlled for, the effect of living alone considerably attenuated and disappeared for ages 80–89 years. Important unobserved time-invariant factors could consist of older Finnish adults’ preference of independence and autonomy [26], and individual physiological and psychological reserves and resilience which influence their capacity to cope and recover from stressful life events (e.g., widowhood and bereavement) [40]. Older adults living alone may also develop better coping mechanisms and contingency plans by using formal health services regularly, extending their social networks actively, and participating in social activities frequently [16, 41]. There could also be individual characteristics, such as health status and SES in early-life, that are highly associated with being selected into marriage or cohabitation at young ages and staying in partnership and maintaining good health status at older ages [37].

The large gender differences observed in the logistic regression models were not replicated in the individual LPM-FE model, which may link to female advantages in physiological reserve [42] and in coping with widowhood [43, 44]. Furthermore, younger men with anti-social behaviour [45] and problem drinking [46, 47] are less likely to be selected into partnership. This selection earlier in life may partly explain the large reduction in the effect of living alone among young-old (< 70 years) men in the LPM-FE model. The increased probability of heavy hospital care use found among young-old women in the LPM-FE model may relate to worse consequences of widowhood for younger than older widows [48].

### Co-residing with children

In Finland, co-residence with adult children typically decreases with age as offspring form their own households. Co-residence with adult children may bring unique stress and undermine older parents’ health [21, 49, 50]. A recent study showed that having adult children moving back home was associated with poorer quality of life among Europeans aged 50–75 years, using linear individual fixed-effects regression [30]. We did not find strong evidence of a detrimental effect of co-residing with adult children on hospital care use in our LPM-FE models. Adult children living with their parents tend to be unmarried, unemployed, and less educated than those living independently [18]. We do not have direct information on adult children’s characteristics. It is nevertheless possible that those adult children may never have left their parental home due to health or other problems, or they may have moved back to seek help. The lack of association between living with adult children and hospital care use at ages 80–89 years for both genders, regardless of the presence of the partner, may reflect the alternative possibility that the elderly’s health declines to a large extent and then their adult children move back to take care of them rather than vice versa.

Additional analyses focussed on co-residence with minor children. In contrast to a previous Finnish study on younger adults (30–64 years old) reported a lower risk of mortality among those living with minor children [7], we found a detrimental effect of co-residing with minor children among both men and women in the LPM-FE models, particularly at ages 60–69 years for men. This could be due to several stressors that parents are exposed to, such as daily demands and time constraints of parenting, increased strain between parents, and work-family conflict [21], which may be worse for old fathers who postponed their parenthood.

### Living with other persons

Americans aged 65 years and older living with other persons, either related or unrelated, were shown to have poorest health, even worse than those living alone [19]. This is consistent with our findings from logistic regression models. Unfortunately, in our data we could not distinguish living with relatives (other than those belonging to the nuclear family) or non-relatives (e.g., friends). However, in the LPM-FE models, living with other persons seemed to be protective among old-old women although the effect did not reach statistical significance. This may suggest that young-old women living with other persons are more likely to be caregivers, whereas at old-old ages they are more likely to be care recipients who need assistance and care from others but do not receive them from their partner or children.

### Strengths and limitations

Our study has several important strengths. Our findings were drawn from rich data of a very large national representative sample of middle-aged and older men and women residing in Finland in a 20-year period. We provided fresh evidence not only on the relationship between living alone and hospital care use, but also less studied yet not uncommon living arrangements such as living with minor and adult children and with persons other than family members. Both living arrangements and hospital care use were extracted from national registers that are less prone to measurement error than self-reported measures often used in surveys. Additionally, the register-based sample does not suffer from non-response bias or attrition as do surveys. The sufficient within-individual changes in our data enabled us to apply the individual LPM-FE technique.

We also acknowledge that there are some limitations. The LPM-FE does not control for time-varying unobserved individual factors [28]. We reported relative difference from LPM-FE models rather than absolute difference (model coefficients, see Additional files 2-3) in order to keep the results comparable to those from logistic regression. The discrepancy of the age pattern of the relationship between living arrangements and hospital care use when using relative difference vs. absolute difference is not unexpected as the relative difference is largely determined by the level of hospital care use in the reference group [51]. Furthermore, LPM-FE does not use information from study subjects whose living arrangement remained the same in the 10-year observation window (21–28% in our sample) [28]. The characteristics of study subjects whose living arrangement did not change nevertheless were very similar to those whose living arrangement changed (see Additional file 8). We additionally repeated the analysis using linear probability model in the LPM-FE sample (see Additional file 9), and the relative differences were slightly smaller than those in the full sample. The sample exclusion in LPM-FE analyses is thus unlikely to significantly bias our results. In addition, we cannot rule out the possibility of reverse causation, that is pre-existing long-lasting conditions and disability could affect both an individual’s hospital care use and living arrangement (e.g., leading to living alone). Caregiving in the family may also play a role. For instance, living with a partner with ill-health may not necessarily bring health benefits, but due to the heavy caring responsibilities it may be detrimental to health. With no information on caregiving, we cannot separate the possible adverse effect of taking care of an older person on the caregiver’s health. Furthermore, our data was relatively old and lacked measurement of key mediating mechanisms. For example, the relationship between living arrangements other than living with a partner and health may be extensively mitigated by social ties from family and friends that older adults have [24, 26]. Formal investigations on the possible biological, behavioural, and psychosocial pathways linking living arrangements and health at older ages are needed, as well as those evaluating the possible health effects of co-residence with other persons than family members and the characteristics of adult children living in the same household. Further research of cause-specific hospital care use (e.g., due to external causes, cardiovascular disease, cancer, diabetes or other chronic diseases) could also provide more information on the mechanisms.

Last but not least, logistic regression and linear probability models give total population-average effects; whereas the individual linear probability fixed-effects regression provides subject-specific estimates for the sub-population that experienced a change in living arrangements [28]. Due to our study design, effects of living arrangements estimated using fixed-effects models are relatively short-term: they estimate the change in hospital care use after living arrangement change within a 10-year observation window. Accordingly, our findings from the logistic regression models are important and relevant for policies tailored for older adults with certain types of living arrangement to reduce hospital care use in the whole population.

## Conclusion

Middle-aged and older men and women living alone or living with persons other than family members in Finland were more likely to be heavy users of hospital care than those living only with their partner; these effects were larger among men than among women and decreased with age. Co-residence with adult children increased the odds of heavy hospital care use at ages 60–79 years. A significant part of these associations was driven by observed socioeconomic status. Additional adjustment for unobserved time-invariant individual characteristics attenuated these associations further. Despite all these adjustments, a moderate adverse effect of living alone remained. These findings underscore the importance for further studies using longitudinal data to fully evaluate individual social and behavioural characteristics that underlie the relationship between living arrangements and health at older ages.

## Additional files

Additional file 1: Socio-demographic characteristics of analytical sample. (DOCX 18 kb)

Additional file 2: Linear probability model coefficients and predicted probability among men, by 10-year age groups. (DOCX 18 kb)

Additional file 3: Linear probability model coefficients and predicted probability among women, by 10-year age groups. (DOCX 18 kb)

Additional file 4: Risk of heavy hospital care use for men and women living with the partner and at least one minor child. (DOCX 138 kb)

Additional file 5: Risk of heavy hospital care use for lone fathers and lone mothers living with at least one minor child. (DOCX 139 kb)

Additional file 6: Living arrangements and risk of having 5 or more hospital days per hospitalisation episode in a year among men, by 10-year age groups. (DOCX 21 kb)

Additional file 7: Living arrangements and risk of having 5 or more hospital days per hospitalisation episode in a year among women, by 10-year age groups. (DOCX 20 kb)

Additional file 8: Socio-demographic characteristics of samples with living arrangement unchanged vs. living arrangement changed. (DOCX 21 kb)

Additional file 9: Living arrangements and risk of being hospitalised for 8 or more days in a year using linear probability model among study subjects whose living arrangement changed, by gender and 10-year age groups. (DOCX 19 kb)

## Data Availability

The data that support the findings of this study are available from the Statistics Finland and the National Institute for Health and Welfare of Finland but restrictions apply to the availability of these data, which were used under license for the current study, and so are not publicly available.

## Abbreviations

FE: Fixed-effects
LPM: Linear probability model
OLS: Ordinary least squares
SES: Socioeconomic status
