# Supplementary material for: The relationship between living arrangements and higher use of hospital care at middle and older ages: to what extent do observed and unobserved individual characteristics explain this association?
Source: BMC Public Health. 2019 Jul 29;19:1011. doi: 10.1186/s12889-019-7296-x (PMC6664712; doi:10.1186/s12889-019-7296-x)
Supplement: Supplementary file 1 — Socio-demographic characteristics of analytical sample. (DOCX 18 kb) [file 12889_2019_7296_MOESM1_ESM.docx]

## Additional file 1. Socio-demographic characteristics of analytical sample

|  | **Men (%)** | | | | **Women (%)** | | | |
| --- | --- | --- | --- | --- | --- | --- | --- | --- |
|  | **50-59 years** | **60-69 years** | **70-79 years** | **80-89 years** | **50-59 years** | **60-69 years** | **70-79 years** | **80-89 years** |
| **Total number of observations** | 683,709 | 479,938 | 282,142 | 89,798 | 702,204 | 569,573 | 457,226 | 216,814 |
| **Region of residence** |  |  |  |  |  |  |  |  |
| South | 47.5 | 45.1 | 44.0 | 45.8 | 49.3 | 46.5 | 46.0 | 48.0 |
| West | 26.3 | 27.6 | 28..9 | 29.1 | 25.9 | 27.2 | 28.3 | 28.5 |
| East | 14.4 | 15.5 | 15.5 | 14.8 | 13.6 | 15.1 | 15.3 | 14.2 |
| North | 11.9 | 11.9 | 11.6 | 10.3 | 11.2 | 11.1 | 10.4 | 9.4 |
| **Education^§^** |  |  |  |  |  |  |  |  |
| Compulsory only | 46.4 | 64.7 | 74.5 | 77.5 | 46.0 | 67.1 | 78.6 | 82.7 |
| Upper secondary | 29.6 | 17.8 | 11.9 | 9.9 | 31.4 | 20.2 | 13.7 | 10.7 |
| Tertiary | 24.0 | 17.6 | 13.6 | 12.6 | 22.6 | 12.7 | 7.7 | 6.6 |
| **Household income tertile^§^** |  |  |  |  |  |  |  |  |
| Low | 33.3 | 33.4 | 33.5 | 33.5 | 33.4 | 33.4 | 33.7 | 33.7 |
| Middle | 33.4 | 33.2 | 33.2 | 33.2 | 33.4 | 33.3 | 33.1 | 33.1 |
| High | 33.3 | 33.3 | 33.3 | 33.3 | 33.3 | 33.3 | 33.2 | 33.2 |
| **Labour force status^§^** |  |  |  |  |  |  |  |  |
| Employed | 68.4 | 16.4 | 0.4 | 0 | 69.6 | 13.2 | 0.3 | 0 |
| Unemployed | 9.9 | 2.4 | 0 | 0 | 9.5 | 2.4 | 0 | 0 |
| Pensioners | 18.6 | 79.4 | 99.5 | 100.0 | 15.9 | 81.2 | 99.6 | 100.0 |
| Other | 3.1 | 1.8 | 0.1 | 0 | 5.0 | 3.2 | 0.1 | 0 |
| **Marital status^§^** |  |  |  |  |  |  |  |  |
| Married | 70.2 | 75.1 | 73.2 | 58.2 | 66.1 | 57.2 | 35.7 | 13.7 |
| Unmarried | 13.7 | 10.2 | 7.5 | 5.6 | 9.9 | 9.4 | 10.5 | 11.9 |
| Divorced | 14.5 | 10.2 | 6.1 | 3.7 | 17.1 | 12.3 | 8.2 | 5.7 |
| Widowed | 1.6 | 4.6 | 13.2 | 32.4 | 6.9 | 21.1 | 45.6 | 68.6 |

**^§^** Education, household income, labour force status, and marital status were measured at the time when the study subjects entered into the age groups.
