# Supplementary material for: The relationship between living arrangements and higher use of hospital care at middle and older ages: to what extent do observed and unobserved individual characteristics explain this association?
Source: BMC Public Health. 2019 Jul 29;19:1011. doi: 10.1186/s12889-019-7296-x (PMC6664712; doi:10.1186/s12889-019-7296-x)
Supplement: Supplementary file 2 — Linear probability model coefficients and predicted probability among men, by 10-year age groups. (DOCX 18 kb) [file 12889_2019_7296_MOESM2_ESM.docx]

Additional file 2. Linear probability model coefficients and predicted probability among men, by 10-year age groups

|  | **LPM** | | **LPM-FE** | |
| --- | --- | --- | --- | --- |
|  | Coefficient (95% CI) | Predicted probability (95% CI) | Coefficient (95% CI) | Predicted probability (95% CI) |
| **50-59 years** |  |  |  |  |
| Living with a partner only | Ref | 0.049 (0.047, 0.050) | Ref | 0.050 (0.048, 0.051) |
| Living with a partner & 1+ minor child | -0.007 (-0.009, -0.005) | 0.042 (0.040, 0.043) | 0.005 (0.002, 0.008) | 0.054 (0.052, 0.057) |
| Living with a partner & adult children | -0.002 (-0.004, -0.001) | 0.046 (0.045, 0.048) | 0.003 (0.001, 0.005) | 0.053 (0.051, 0.054) |
| Lone parent living with 1+ minor child | -0.003 (-0.010, 0.005) | 0.046 (0.039, 0.053) | 0.123 (0.003, 0.023) | 0.063 (0.053, 0.072) |
| Lone parent living with adult children | 0.005 (-0.001, 0.011) | 0.054 (0.048, 0.060) | 0.010 (0.003, 0.016) | 0.060 (0.053, 0.066) |
| Living alone | 0.005 (0.019, 0.025) | 0.071 (0.068, 0.073) | 0.007 (0.004, 0.011) | 0.057 (0.054, 0.060) |
| Living with others | 0.025 (0.020, 0.030) | 0.073 (0.069, 0.078) | 0.000 (-0.006, 0.005) | 0.050 (0.044, 0.054) |
| Other | 0.022 (0.016, 0.029) | 0.071 (0.065, 0.078) | -0.009 (-0.016, -0.003) | 0.040 (0.034, 0.047) |
| **60-69 years** |  |  |  |  |
| Living with a partner only | Ref | 0.092 (0.090, 0.094) | Ref | 0.097 (0.095, 0.099) |
| Living with a partner & 1+ minor child | 0.005 (-0.002, 0.012) | 0.097 (0.090, 0.104) | 0.021 (0.011, 0.031) | 0.118 (0.109, 0.127) |
| Living with a partner & adult children | 0.006 (0.003, 0.010) | 0.098 (0.095, 0.102) | 0.006 (0.001, 0.010) | 0.103 (0.099, 0.107) |
| Lone parent living with 1+ minor child | 0.020 (-0.019, 0.059) | 0.112 (0.073, 0.151) | 0.050 (0.011, 0.089) | 0.147 (0.108, 0.186) |
| Lone parent living with adult children | 0.015 (0.003, 0.027) | 0.107 (0.095, 0.118) | 0.002 (-0.011, 0.015) | 0.099 (0.086, 1.112) |
| Living alone | 0.030 (0.025, 0.035) | 0.122 (0.117, 0.126) | 0.010 (0.004, 0.016) | 0.107 (0.102, 0.112) |
| Living with others | 0.025 (0.017, 0.033) | 0.117 (0.110, 0.125) | 0.006 (-0.004, 0.015) | 0.103 (0.009, 0.111) |
| Other | 0.117 (0.096, 0.136) | 0.209 (0.190, 0.228) | -0.019 (-0.033, -0.006) | 0.078 (0.064, 0.091) |
| **70-79 years** |  |  |  |  |
| Living with a partner only | Ref | 0.190 (0.187, 0.194) | Ref | 0.196 (0.093, 0.199) |
| Living with a partner & adult children | 0.015 (0.007, 0.023) | 0.206 (0.198, 0.214) | 0.002 (-0.010, 0.013) | 0.198 (0.187, 0.209) |
| Lone parent living with adult children | 0.012 (-0.008, 0.031) | 0.202 (0.184, 0.220) | 0.022 (0.000, 0.044) | 0.218 (0.197, 0.239) |
| Living alone | 0.020 (0.009, 0.031) | 0.211 (0.202, 0.219) | 0.017 (0.008, 0.026) | 0.213 (0.206, 0.220) |
| Living with others | 0.017 (0.001, 0.032) | 0.207 (0.194, 0.221) | 0.006 (-0.010, 0.023) | 0.202 (0.187, 0.218) |
| Other | 0.231 (0.206, 0.256) | 0.421 (0.397, 0.445) | -0.084 (-0.101, -0.067) | 0.112 (0.096, 0.128) |
| **80-89 years** |  |  |  |  |
| Living with a partner only | Ref | 0.318 (0.308, 0.328) | Ref | 0.342 (0.335, 0.350) |
| Living with a partner & adult children | -0.016 (-0.036, 0.003) | 0.302 (0.281, 0.322) | -0.023 (-0.058, 0.012) | 0.319 (0.286, 0.352) |
| Lone parent living with adult children | 0.031 (-0.001, 0.063) | 0.349 (0.321, 0.377) | 0.023 (-0.021, 0.067) | 0.366 (0.324, 0.407) |
| Living alone | 0.033 (0.013, 0.054) | 0.351 (0.338, 0.364) | 0.006 (-0.009, 0.021) | 0.348 (0.338, 0.358) |
| Living with others | 0.066 (0.040, 0.092) | 0.384 (0.363, 0.405) | 0.012 (-0.015, 0.038) | 0.354 (0.331, 0.377) |
| Other | 0.118 (0.091, 0.144) | 0.436 (0.413, 0.458) | -0.160 (-0.181, -0.140) | 0.182 (0.164, 0.200) |

LPM: linear probability model, adjusting for all covariates in Model 3

LPM-FE: linear probability model with fixed-effects

CI: confidence interval; Ref: reference category

Model 3: adjusting for current age dummies, region of residence, education, household income, labour force status, and marital status at time of entry to the age group
