# Supplementary material for: The relationship between living arrangements and higher use of hospital care at middle and older ages: to what extent do observed and unobserved individual characteristics explain this association?
Source: BMC Public Health. 2019 Jul 29;19:1011. doi: 10.1186/s12889-019-7296-x (PMC6664712; doi:10.1186/s12889-019-7296-x)
Supplement: Supplementary file 3 — Linear probability model coefficients and predicted probability among women, by 10-year age groups. (DOCX 18 kb) [file 12889_2019_7296_MOESM3_ESM.docx]

Additional file 3. Linear probability model coefficients and predicted probability among women, by 10-year age groups

|  | **LPM** | | **LPM-FE** | |
| --- | --- | --- | --- | --- |
|  | Coefficient (95% CI) | Predicted probability (95% CI) | Coefficient (95% CI) | Predicted probability (95% CI) |
| **50-59 years** |  |  |  |  |
| Living with a partner only | Ref | 0.044 (0.043, 0.045) | Ref | 0.044 (0.042, 0.045) |
| Living with a partner & 1+ minor child | -0.004 (-0.006, -0.002) | 0.040 (0.038, 0.042) | 0.004 (0.001, 0.007) | 0.047 (0.045, 0.050) |
| Living with a partner & adult children | -0.001 (-0.002, 0.001) | 0.043 (0.042, 0.045) | 0.003 (0.001, 0.005) | 0.047 (0.045, 0.048) |
| Lone parent living with 1+ minor child | -0.003 (-0.008, 0.001) | 0.041 (0.037, 0.045) | 0.007 (0.001, 0.013) | 0.050 (0.045, 0.056) |
| Lone parent living with adult children | 0.002 (-0.001, 0.006) | 0.046 (0.043, 0.050) | 0.006 (0.002, 0.010) | 0.050 (0.046, 0.053) |
| Living alone | 0.009 (0.007, 0.012) | 0.053 (0.051, 0.055) | 0.006 (0.003, 0.009) | 0.049 (0.047, 0.052) |
| Living with others | 0.013 (0.007, 0.019) | 0.057 (0.051, 0.062) | 0.001 (-0.004, 0.007) | 0.045 (0.040, 0.050) |
| Other | 0.039 (0.027, 0.050) | 0.083 (0.071, 0.094) | -0.007 (-0.017, 0.002) | 0.036 (0.027, 0.045) |
| **60-69 years** |  |  |  |  |
| Living with a partner only | Ref | 0.074 (0.071, 0.075) | Ref | 0.076 (0.074, 0.078) |
| Living with a partner & 1+ minor child | -0.013 (-0.028, 0.030) | 0.061 (0.046, 0.077) | 0.010 (-0.009, 0.289) | 0.086 (0.067, 0.105) |
| Living with a partner & adult children | 0.006 (0.002, 0.009) | 0.079 (0.076, 0.083) | 0.002 (-0.002, 0.007) | 0.078 (0.074, 0.083) |
| Lone parent living with 1+ minor child | -0.002 (-0.037, 0.033) | 0.072 (0.037, 0.107) | 0.016 (-0.022, 0.053) | 0.092 (0.054, 0.129) |
| Lone parent living with adult children | 0.008 (0.002, 0.013) | 0.082 (0.077, 0.086) | 0.006 (-0.005, 0.013) | 0.082 (0.076, 0.088) |
| Living alone | 0.009 (0.006, 0.013) | 0.083 (0.080, 0.085) | 0.008 (0.004, 0.013) | 0.084 (0.082, 0.087) |
| Living with others | 0.020 (0.013, 0.026) | 0.093 (0.088, 0.099) | 0.004 (-0.004, 0.011) | 0.080 (0.073, 0.086) |
| Other | 0.170 (0.144, 0.196) | 0.244 (0.218, 0.269) | -0.034 (-0.047, -0.021) | 0.042 (0.029, 0.055) |
| **70-79 years** |  |  |  |  |
| Living with a partner only | Ref | 0.161 (0.155, 0.168) | Ref | 0.173 (0.169, 0.177) |
| Living with a partner & adult children | 0.011 (0.002, 0.020) | 0.172 (0.162, 0.183) | -0.007 (-0.020, 0.005) | 0.166 (0.154, 0.178) |
| Lone parent living with adult children | 0.016 (0.004, 0.027) | 0.177 (0.170, 03184) | 0.013 (0.002, 0.024) | 0.186 (0.176, 0.196) |
| Living alone | 0.012 (0.002, 0.022) | 0.173 (0.169, 0.177) | 0.007 (0.001, 0.013) | 0.180 (0.178, 0.183) |
| Living with others | 0.037 (0.026, 0.049) | 0.199 (0.191, 0.206) | -0.003 (-0.012, 0.007) | 0.170 (0.163, 0.178) |
| Other | 0.238 (0.220, 0.257) | 0.399 (0.383, 0.416) | -0.094 (-0.105, -0.083) | 0.080 (0.070, 0.089) |
| **80-89 years** |  |  |  |  |
| Living with a partner only | Ref | 0.313 (0.295, 0.330) | Ref | 0.334 (0.323, 0.345) |
| Living with a partner & adult children | -0.016 (-0.043, 0.011) | 0.297 (0.266, 0.328) | -0.007 (-0.047, 0.033) | 0.327 (0.289, 0.365) |
| Lone parent living with adult children | -0.008 (-0.031, 0.014) | 0.304 (0.293, 0.315) | -0.003 (-0.037, 0.020) | 0.331 (0.312, 0.350) |
| Living alone | 0.002 (-0.018, 0.023) | 0.315 (0.301, 0.319) | 0.010 (-0.002, 0.023) | 0.344 (0.341, 0.348) |
| Living with others | 0.037 (0.015, 0.059) | 0.350 (0.341, 0.359) | -0.007 (-0.023, 0.009) | 0.327 (0.316, 0.337) |
| Other | 0.087 (0.065, 0.109) | 0.400 (0.388, 0.411) | -0.188 (-0.202, -0.173) | 0.146 (0.138, 0.155) |

LPM: linear probability model, adjusting for all covariates in Model 3

LPM-FE: linear probability model with fixed-effects

CI: confidence interval; Ref: reference category

Model 3: adjusting for current age dummies, region of residence, education, household income, labour force status, and marital status at time of entry to the age group
