# Supplementary material for: The relationship between living arrangements and higher use of hospital care at middle and older ages: to what extent do observed and unobserved individual characteristics explain this association?
Source: BMC Public Health. 2019 Jul 29;19:1011. doi: 10.1186/s12889-019-7296-x (PMC6664712; doi:10.1186/s12889-019-7296-x)
Supplement: Supplementary file 5 — Risk of heavy hospital care use for lone fathers and lone mothers living with at least one minor child. (DOCX 139 kb) [file 12889_2019_7296_MOESM5_ESM.docx]

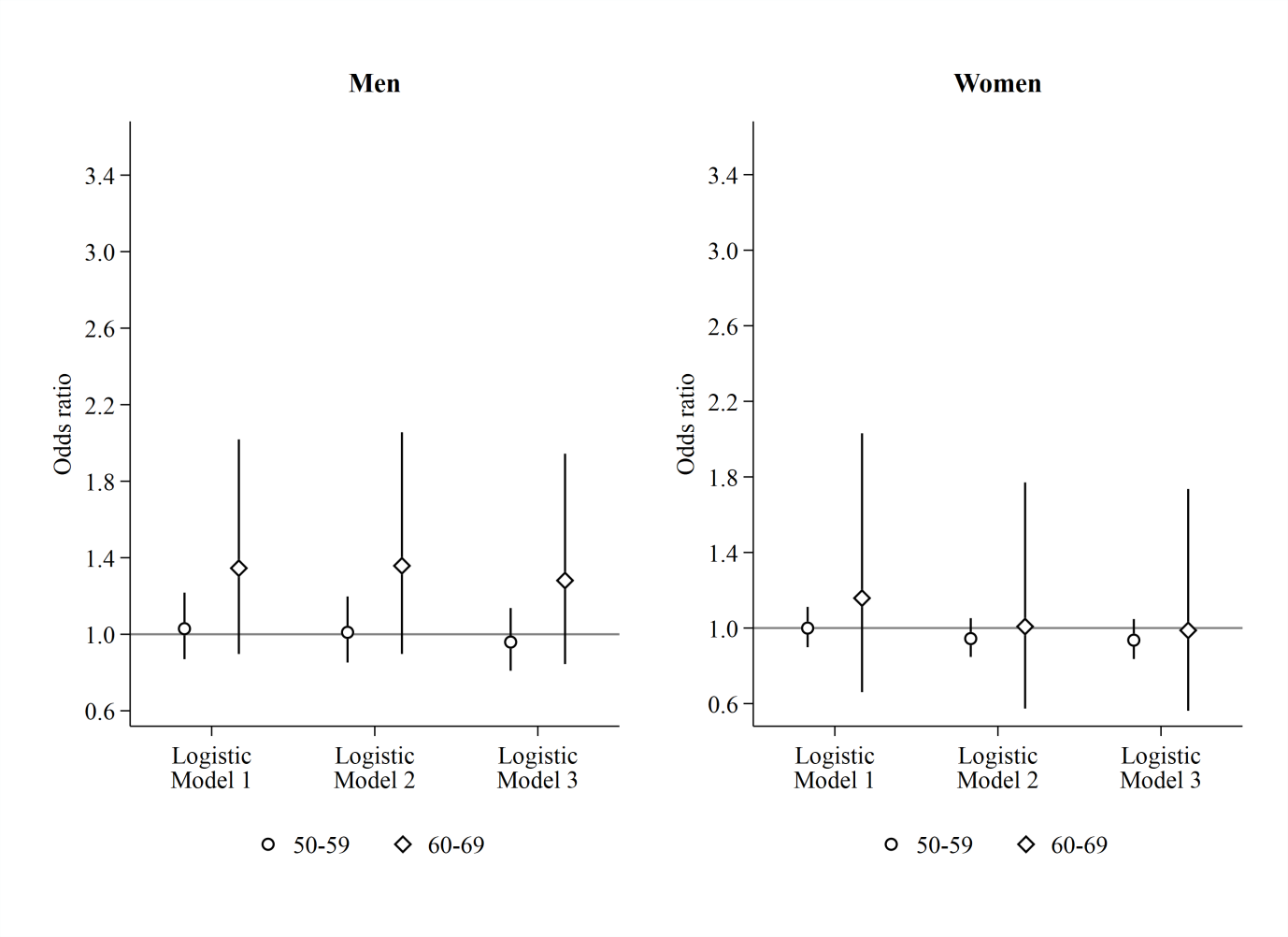


Reference group: living with the partner only

Logistic Model 1: adjusting for time-varying current age dummies and region of residence

Logistic Model 2: Model 1 + education, household income, and labour force status measured at the time when study

subjects entered into the current age group

Logistic Model 3: Model 2 + marital status measured at the time when study subjects entered into the current age group

Additional file 5. Risk of heavy hospital care use for lone fathers and lone mothers living with at least one minor child
