# Supplementary material for: The relationship between living arrangements and higher use of hospital care at middle and older ages: to what extent do observed and unobserved individual characteristics explain this association?
Source: BMC Public Health. 2019 Jul 29;19:1011. doi: 10.1186/s12889-019-7296-x (PMC6664712; doi:10.1186/s12889-019-7296-x)
Supplement: Supplementary file 6 — Living arrangements and risk of having 5 or more hospital days per hospitalisation episode in a year among men, by 10-year age groups. (DOCX 21 kb) [file 12889_2019_7296_MOESM6_ESM.docx]

## Additional file 6. Living arrangements and risk of having 5 or more hospital days per hospitalisation episode in a year among men, by 10-year age groups

|  | **Logistic Model 1** | **Logistic Model 2** | **Logistic Model 3** | **LPM** | **LPM-FE** |
| --- | --- | --- | --- | --- | --- |
|  | OR (95% CI) | OR (95% CI) | OR (95% CI) | Relative difference (95% CI) | Relative difference (95% CI) |
| **50-59 years** |  |  |  |  |  |
| Living with a partner only | Ref | Ref | Ref | Ref | Ref |
| Living with a partner & 1+ minor child | 0.81 (0.78, 0.85) | 0.85 (0.81, 0.89) | 0.85 (0.71, 0.89) | 0.89 (0.85, 0.92) | 1.05 (0.99, 1.11) |
| Living with a partner & adult children | 0.94 (0.90, 0.97) | 0.97 (0.93, 1.00) | 0.97 (0.93, 1.00) | 0.98 (0.94, 1.01) | 1.03 (0.99, 1.07) |
| Lone parent living with 1+ minor child | 1.05 (0.89, 1.23) | 1.04 (0.88, 1.23) | 0.99 (0.84, 1.17) | 0.98 (0.73, 1.13) | 1.27 (1.07, 1.46) |
| Lone parent living with adult children | 1.22 (1.11, 1.35) | 1.19 (1.07, 1.32) | 1.13 (1.01, 1.25) | 1.11 (0.99, 1.22) | 1.20 (1.07, 1.33) |
| Living alone | 1.69 (1.62, 1.75) | 1.43 (1.38, 1.48) | 1.47 (1.40, 1.54) | 1.46 (1.40, 1.52) | 1.15 (1.08, 1.23) |
| Living with others | 1.80 (1.70, 1.92) | 1.40 (1.32, 1.49) | 1.48 (1.38, 1.59) | 1.50 (1.40, 1.60) | 0.96 (0.86, 1.07) |
| Other | 1.77 (1.64, 1.92) | 1.36 (1.26, 1.48) | 1.50 (1.37, 1.64) | 1.49 (1.36, 1.61) | 0.91 (0.78, 1.04) |
| **60-69 years** |  |  |  |  |  |
| Living with a partner only | Ref | Ref | Ref | Ref | Ref |
| Living with a partner & 1+ minor child | 1.01 (0.92, 1.10) | 1.07 (0.97, 1.17) | 1.06 (0.97, 1.17) | 1.06 (0.98, 1.13) | 1.23 (1.13, 1.34) |
| Living with a partner & adult children | 1.09 (1.05, 1.13) | 1.09 (1.05, 1.14) | 1.09 (1.05, 1.14) | 1.08 (1.05, 1.12) | 1.06 (1.01, 1.10) |
| Lone parent living with 1+ minor child | 1.24 (0.84, 1.84) | 1.25 (0.85, 1.83) | 1.19 (0.80, 1.75) | 1.14 (0.77, 1.51) | 1.36 (0.95, 1.76) |
| Lone parent living with adult children | 1.28 (1.14, 1.43) | 1.24 (1.11, 1.39) | 1.19 (1.06, 1.34) | 1.16 (1.03, 1.28) | 1.07 (0.93, 1.21) |
| Living alone | 1.51 (1.46, 1.56) | 1.39 (1.35, 1.44) | 1.36 (1.30, 1.43) | 1.33 (1.27, 1.38) | 1.15 (1.08, 1.21) |
| Living with others | 1.42 (1.34, 1.51) | 1.31 (1.23, 1.39) | 1.31 (1.22, 1.41) | 1.28 (1.19, 1.36) | 1.06 (0.97, 1.16) |
| Other | 2.80 (2.51, 3.12) | 2.49 (2.24, 2.78) | 2.50 (2.22, 2.80) | 2.23 (2.03, 2.44) | 0.93 (0.79, 1.07) |
| **70-79 years** |  |  |  |  |  |
| Living with a partner only | Ref | Ref | Ref | Ref | Ref |
| Living with a partner & adult children | 1.14 (1.08, 1.20) | 1.13 (1.08, 1.19) | 1.14 (1.08, 1.19) | 1.10 (1.06, 1.15) | 1.03 (0.97, 1.09) |
| Lone parent living with adult children | 1.18 (1.08, 1.30) | 1.18 (1.07, 1.30) | 1.14 (1.03, 1.26) | 1.08 (0.97, 1.18) | 1.08 (0.97, 1.20) |
| Living alone | 1.27 (1.23, 1.31) | 1.23 (1.19, 1.27) | 1.21 (1.16, 1.27) | 1.14 (1.08, 1.20) | 1.12 (1.07, 1.17) |
| Living with others | 1.21 (1.13, 1.30) | 1.17 (1.09, 1.25) | 1.19 (1.10, 1.29) | 1.12 (1.04, 1.20) | 1.03 (0.94, 1.11) |
| Other | 3.12 (2.83, 3.44) | 3.00 (2.72, 3.31) | 2.99 (2.71, 3.31) | 2.20 (2.06, 2.34) | 0.63 (0.55, 0.72) |
| **80-89 years** |  |  |  |  |  |
| Living with a partner only | Ref | Ref | Ref | Ref | Ref |
| Living with a partner & adult children | 0.97 (0.89, 1.06) | 0.96 (0.88, 1.06) | 0.96 (0.88, 1.06) | 0.97 (0.81, 1.03) | 0.98 (0.87, 1.08) |
| Lone parent living with adult children | 1.07 (0.95, 1.20) | 1.06 (0.94, 1.19) | 1.06 (0.94, 1.21) | 1.11 (1.01, 1.21) | 1.04 (0.91, 1.17) |
| Living alone | 1.09 (1.04, 1.14) | 1.07 (1.03, 1.12) | 1.11 (1.04, 1.18) | 1.13 (1.07, 1.20) | 1.03 (0.98, 1.07) |
| Living with others | 1.23 (1.14, 1.34) | 1.22 (1.12, 1.32) | 1.28 (1.16, 1.41) | 1.24 (1.15, 1.33) | 1.06 (0.98, 1.13) |
| Other | 1.66 (1.51, 1.83) | 1.63 (1.48, 1.79) | 1.67 (1.51, 1.84) | 1.44 (1.34, 1.53) | 0.58 (0.52, 0.63) |

LPM: linear probability model, adjusting for all covariates in Model 3

LPM-FE: linear probability model with fixed-effects

OR: odds ratio; CI: confidence interval; Ref: reference category

Model 1: adjusting for current age dummies, region of residence;

Model 2: Model 1 + education, household income, and labour force status at time of entry to the age group;

Model 3: Model 2 + marital status at time of entry to the age group
