# Supplementary material for: The relationship between living arrangements and higher use of hospital care at middle and older ages: to what extent do observed and unobserved individual characteristics explain this association?
Source: BMC Public Health. 2019 Jul 29;19:1011. doi: 10.1186/s12889-019-7296-x (PMC6664712; doi:10.1186/s12889-019-7296-x)
Supplement: Supplementary file 7 — Living arrangements and risk of having 5 or more hospital days per hospitalisation episode in a year among women, by 10-year age groups. (DOCX 20 kb) [file 12889_2019_7296_MOESM7_ESM.docx]

## Additional file 7. Living arrangements and risk of having 5 or more hospital days per hospitalisation episode in a year among women, by 10-year age groups

|  | **Logistic Model 1** | **Logistic Model 2** | **Logistic Model 3** | **LPM** | **LPM-FE** |
| --- | --- | --- | --- | --- | --- |
|  | OR (95% CI) | OR (95% CI) | OR (95% CI) | Relative difference (95% CI) | Relative difference (95% CI) |
| **50-59 years** |  |  |  |  |  |
| Living with a partner only | Ref | Ref | Ref | Ref | Ref |
| Living with a partner & 1+ minor child | 0.86 (0.82, 0.91) | 0.92 (0.87, 0.97) | 0.92 (0.87, 0.97) | 0.94 (0.89, 0.98) | 1.07 (1.01, 1.14) |
| Living with a partner & adult children | 0.98 (0.94, 1.01) | 1.00 (0.97, 1.04) | 1.00 (0.97, 1.04) | 1.01 (0.97, 1.04) | 1.05 (1.01, 1.10) |
| Lone parent living with 1+ minor child | 1.00 (0.91, 1.10) | 0.96 (0.87, 1.06) | 0.96 (0.87, 1.07) | 0.96 (0.87, 1.05) | 1.04 (0.91, 1.17) |
| Lone parent living with adult children | 1.19 (1.12, 1.27) | 1.09 (1.02, 1.16) | 1.10 (1.02, 1.17) | 1.09 (1.01, 1.16) | 1.08 (0.99, 1.17) |
| Living alone | 1.29 (1.25, 1.34) | 1.18 (1.14, 1.23) | 1.20 (1.14, 1.26) | 1.19 (1.14, 1.24) | 1.07 (0.99, 1.13) |
| Living with others | 1.63 (1.50, 1.76) | 1.24 (1.15, 1.34) | 1.28 (1.18, 1.40) | 1.30 (1.19, 1.42) | 1.06 (0.94, 1.18) |
| Other | 2.34 (2.06, 2.66) | 1.58 (1.39, 1.80) | 1.68 (1.47, 1.92) | 1.76 (1.52, 2.00) | 0.76 (0.56, 0.96) |
| **60-69 years** |  |  |  |  |  |
| Living with a partner only | Ref | Ref | Ref | Ref | Ref |
| Living with a partner & 1+ minor child | 0.80 (0.58, 1.11) | 0.81 (0.58, 1.11) | 0.81 (0.58, 1.11) | 0.86 (0.66, 1.05) | 1.06 (0.81, 1.30) |
| Living with a partner & adult children | 1.08 (1.03, 1.13) | 1.08 (1.03, 1.13) | 1.08 (1.03, 1.13) | 1.07 (1.03, 1.11) | 1.02 (0.97, 1.08) |
| Lone parent living with 1+ minor child | 0.98 (0.53, 1.79) | 0.85 (0.47, 1.56) | 0.83 (0.46, 1.52) | 0.84 (0.40, 1.28) | 1.05 (0.57, 1.52) |
| Lone parent living with adult children | 1.22 (1.15, 1.29) | 1.14 (1.08, 1.21) | 1.11 (1.04, 1.19) | 1.10 (1.03, 1.17) | 1.09 (1.01, 1.17) |
| Living alone | 1.20 (1.17, 1.24) | 1.14 (1.11, 1.17) | 1.12 (1.07, 1.17) | 1.11 (1.06, 1.15) | 1.08 (1.03, 1.14) |
| Living with others | 1.38 (1.30, 1.47) | 1.28 (1.21, 1.36) | 1.27 (1.18, 1.36) | 1.25 (1.17, 1.33) | 1.06 (0.97, 1.16) |
| Other | 3.96 (3.47, 4.53) | 3.47 (3.04, 3.97) | 3.46 (3.01, 3.98) | 3.04 (2.72, 3.36) | 0.59 (0.42, 0.76) |
| **70-79 years** |  |  |  |  |  |
| Living with a partner only | Ref | Ref | Ref | Ref | Ref |
| Living with a partner & adult children | 1.12 (1.06, 1.20) | 1.13 (1.06, 1.20) | 1.13 (1.06, 1.20) | 1.10 (1.04, 1.15) | 1.01 (0.94, 1.08) |
| Lone parent living with adult children | 1.18 (1.13, 1.24) | 1.16 (1.11, 1.22) | 1.12 (1.06, 1.18) | 1.11 (1.04, 1.19) | 1.09 (1.03, 1.16) |
| Living alone | 1.15 (1.12, 1.18) | 1.12 (1.09, 1.15) | 1.09 (1.05, 1.13) | 1.08 (1.02, 1.15) | 1.04 (1.01, 1.07) |
| Living with others | 1.35 (1.30, 1.42) | 1.33 (1.28, 1.39) | 1.30 (1.24, 1.37) | 1.26 (1.18, 1.34) | 1.01 (0.95, 1.06) |
| Other | 3.47 (3.24, 3.72) | 3.34 (3.12, 3.58) | 3.27 (3.04, 3.51) | 2.47 (2.33, 2.62) | 0.53 (0.48, 0.59) |
| **80-89 years** |  |  |  |  |  |
| Living with partner only | Ref | Ref | Ref | Ref | Ref |
| Living with partner & adult children | 0.92 (0.80, 1.05) | 0.92 (0.81, 1.06) | 0.92 (0.81, 1.06) | 0.95 (0.86, 1.03) | 0.97 (0.85, 1.09) |
| Lone parent living with adult children | 1.04 (0.98, 1.10) | 1.04 (0.97, 1.10) | 0.99 (0.92, 1.07) | 1.00 (0.93, 1.07) | 0.99 (0.92, 1.05) |
| Living alone | 1.07 (1.03, 1.12) | 1.05 (1.01, 1.09) | 1.01 (0.96, 1.07) | 1.01 (0.95, 1.08) | 1.01 (0.98, 1.05) |
| Living with others | 1.26 (1.20, 1.33) | 1.26 (1.20, 1.33) | 1.22 (1.14, 1.30) | 1.15 (1.07, 1.22) | 0.97 (0.93, 1.02) |
| Other | 1.56 (1.47, 1.66) | 1.52 (1.44, 1.62) | 1.48 (1.38, 1.58) | 1.29 (1.21, 1.38) | 0.48 (0.44, 0.51) |

LPM: linear probability model, adjusting for all covariates in Model 3

LPM-FE: linear probability model with fixed-effects

OR: odds ratio; CI: confidence interval; Ref: reference category

Model 1: adjusting for current age dummies, region of residence;

Model 2: Model 1 + education, household income, and labour force status at time of entry to the age group;

Model 3: Model 2 + marital status at time of entry to the age group
