# Supplementary material for: The relationship between living arrangements and higher use of hospital care at middle and older ages: to what extent do observed and unobserved individual characteristics explain this association?
Source: BMC Public Health. 2019 Jul 29;19:1011. doi: 10.1186/s12889-019-7296-x (PMC6664712; doi:10.1186/s12889-019-7296-x)
Supplement: Supplementary file 8 — Socio-demographic characteristics of samples with living arrangement unchanged vs. living arrangement changed. (DOCX 21 kb) [file 12889_2019_7296_MOESM8_ESM.docx]

## Additional file 8. Socio-demographic characteristics of samples with living arrangement unchanged vs. living arrangement changed

|  | **Living arrangement unchanged** | | | | | | | | **Living arrangement changed** | | | | | | | |
| --- | --- | --- | --- | --- | --- | --- | --- | --- | --- | --- | --- | --- | --- | --- | --- | --- |
|  | **Men (%)** | | | | **Women (%)** | | | | **Men (%)** | | | | **Women (%)** | | | |
|  | **50-59 years** | **60-69 years** | **70-79 years** | **80-89 years** | **50-59 years** | **60-69 years** | **70-79 years** | **80-89 years** | **50-59 years** | **60-69 years** | **70-79 years** | **80-89 years** | **50-59 years** | **60-69 years** | **70-79 years** | **80-89 years** |
| **Total number of observations** | 189,958 | 106,512 | 60,584 | 24,324 | 195,330 | 122,262 | 95,432 | 54,519 | 493,751 | 373,426 | 221,558 | 65,474 | 506,874 | 447,311 | 361,794 | 162,295 |
| **8+ hospital days in a year** |  |  |  |  |  |  |  |  |  |  |  |  |  |  |  |  |
| No | 94.8 | 90.3 | 80.9 | 67.9 | 95.4 | 92.2 | 83.0 | 69.2 | 94.8 | 89.9 | 79.9 | 65.7 | 95.4 | 92.1 | 82.3 | 67.0 |
| Yes | 5.2 | 9.7 | 19.1 | 32.1 | 4.6 | 7.8 | 17.0 | 30.8 | 5.2 | 10.1 | 20.2 | 34.3 | 4.5 | 8.0 | 17.7 | 33.0 |
| **Region of residence** |  |  |  |  |  |  |  |  |  |  |  |  |  |  |  |  |
| South | 47.7 | 45.2 | 43.8 | 44.9 | 49.2 | 46.7 | 45.7 | 47.3 | 47.4 | 45.1 | 44.1 | 46.1 | 49.4 | 46.5 | 46.1 | 48.2 |
| West | 26.0 | 27.3 | 28.5 | 29.1 | 25.8 | 27.0 | 28.0 | 28.4 | 26.4 | 27.5 | 29.0 | 29.1 | 25.9 | 27.3 | 28.4 | 28.6 |
| East | 14.3 | 15.4 | 15.7 | 15.1 | 13.7 | 15.1 | 15.4 | 14.6 | 14.4 | 15.6 | 15.5 | 14.7 | 13.6 | 15.2 | 15.2 | 14.0 |
| North | 11.9 | 12.2 | 12.1 | 10.9 | 11.4 | 11.3 | 10.9 | 9.8 | 11.9 | 11.8 | 11.4 | 10.1 | 11.2 | 11.1 | 10.3 | 9.2 |
| **Education^§^** |  |  |  |  |  |  |  |  |  |  |  |  |  |  |  |  |
| Compulsory only | 45.7 | 63.7 | 74.6 | 78.7 | 45.5 | 66.5 | 79.0 | 83.2 | 46.9 | 65.0 | 74.4 | 77.1 | 46.7 | 67.3 | 78.5 | 82.6 |
| Upper secondary | 29.9 | 18.2 | 12.2 | 9.7 | 31.3 | 20.5 | 13.4 | 10.5 | 29.3 | 17.6 | 11.9 | 10.0 | 31.2 | 20.1 | 13.8 | 10.7 |
| Tertiary | 24.4 | 18.1 | 13.3 | 11.7 | 23.2 | 13.0 | 7.6 | 6.4 | 23.8 | 17.4 | 13.7 | 12.9 | 22.2 | 12.5 | 7.7 | 6.7 |
| **Household income tertile^§^** |  |  |  |  |  |  |  |  |  |  |  |  |  |  |  |  |
| Low | 33.0 | 30.7 | 31.0 | 32.6 | 33.7 | 29.4 | 30.6 | 31.6 | 33.8 | 28.8 | 29.3 | 31.1 | 33.0 | 28.3 | 29.5 | 30.7 |
| Middle | 34.5 | 32.0 | 33.4 | 34.4 | 33.9 | 32.5 | 34.1 | 34.1 | 33.9 | 31.7 | 33.8 | 33.5 | 33.9 | 32.1 | 34.1 | 34.3 |
| High | 32.5 | 37.3 | 35.6 | 33.0 | 32.4 | 38.1 | 35.3 | 34.2 | 32.4 | 39.5 | 36.9 | 35.5 | 33.2 | 39.6 | 36.4 | 35.1 |
| **Labour force status^§^** |  |  |  |  |  |  |  |  |  |  |  |  |  |  |  |  |
| Employed | 78.1 | 33.9 | 0.9 | 0 | 78.4 | 30.1 | 0.6 | 0 | 79.4 | 35.0 | 0.9 | 0 | 79.7 | 31.5 | 0.7 | 0 |
| Unemployed | 6.9 | 7.7 | 0 | 0 | 6.0 | 7..4 | 0 | 0 | 7.1 | 8.4 | 0 | 0 | 6.3 | 8.3 | 0 | 0 |
| Pensioners | 12.1 | 56.6 | 99.1 | 100.0 | 10.5 | 58.1 | 99.3 | 100.0 | 10.6 | 55.1 | 99.0 | 100.0 | 8.9 | 55.6 | 99.3 | 100.0 |
| Other | 2.9 | 1.8 | 0.1 | 0 | 5.1 | 4.5 | 0.1 | 0 | 2.9 | 1.5 | 0.1 | 0 | 5.1 | 4.6 | 0.1 | 0 |
| **Marital status^§^** |  |  |  |  |  |  |  |  |  |  |  |  |  |  |  |  |
| Married | 72.4 | 75.9 | 74.9 | 64.9 | 69.1 | 61.5 | 44.1 | 21.6 | 71.6 | 77.1 | 77.7 | 66.0 | 68.6 | 61.9 | 42.6 | 18.2 |
| Unmarried | 12.7 | 9.8 | 8.1 | 6.0 | 9.0 | 8.7 | 9.9 | 11.5 | 14.5 | 10.4 | 7.4 | 5.5 | 10.4 | 9.6 | 10.6 | 12.1 |
| Divorced | 13.8 | 10.7 | 6.8 | 3.9 | 16.5 | 12.2 | 8.2 | 5.7 | 12.9 | 9.3 | 5.7 | 3.6 | 15.6 | 11.9 | 8.1 | 5.7 |
| Widowed | 1.2 | 3.6 | 10.3 | 25.3 | 5.5 | 17.6 | 37.8 | 61.3 | 1.0 | 3.2 | 9.3 | 25.0 | 5.3 | 16.7 | 38.7 | 64.0 |

**^§^** Education, household income, labour force status, and marital status were measured at the time when the study subjects entered into the age groups.
