# Supplementary material for: The relationship between living arrangements and higher use of hospital care at middle and older ages: to what extent do observed and unobserved individual characteristics explain this association?
Source: BMC Public Health. 2019 Jul 29;19:1011. doi: 10.1186/s12889-019-7296-x (PMC6664712; doi:10.1186/s12889-019-7296-x)
Supplement: Supplementary file 9 — Living arrangements and risk of being hospitalised for 8 or more days in a year using linear probability model among study subjects whose living arrangement changed, by gender and 10-year age groups. (DOCX 19 kb) [file 12889_2019_7296_MOESM9_ESM.docx]

## Additional fie 9. Living arrangements and risk of being hospitalised for 8 or more days in a year using linear probability model among study subjects whose living arrangement changed, by gender and 10-year age groups

|  | **Men** | | | **Women** | | |
| --- | --- | --- | --- | --- | --- | --- |
|  | Coefficient  (95% CI) | Predicted probability  (95% CI) | Relative difference  (95% CI) | Coefficient  (95% CI) | Predicted probability  (95% CI) | Relative difference  (95% CI) |
| **50-59 years** | N=493,751 |  |  | N=506,874 |  |  |
| Living with a partner only | Ref | 0.049 (0.048, 0.050) | Ref | Ref | 0.044 (0.043, 0.046) | Ref |
| Living with a partner & 1+ minor child | -0.007 (-0.009, -0.004) | 0.042 (0.040, 0.044) | 0.86 (0.82, 0.91) | -0.004 (-0.006, -0.001) | 0.041 (0.038, 0.043) | 0.92 (0.86, 0.97) |
| Living with a partner & adult children | -0.002 (-0.004, 0.000) | 0.047 (0.045, 0.049) | 0.96 (0.91, 0.99) | -0.001 (-0.002, 0.001) | 0.044 (0.042, 0.046) | 0.99 (0.94, 1.03) |
| Lone parent living with 1+ minor child | -0.007 (-0.016, 0.002) | 0.042 (0.033, 0.051) | 0.86 (0.67, 1.04) | -0.005 (-0.010, 0.000) | 0.039 (0.034, 0.044) | 0.89 (0.77, 1.01) |
| Lone parent living with adult children | 0.003 (-0.004, 0.011) | 0.052 (0.045, 0.060) | 1.07 (0.91, 1.23) | 0.000 (-0.004, 0.004) | 0.045 (0.041, 0.048) | 1.01 (0.91, 1.10) |
| Living alone | 0.022 (0.018, 0.025) | 0.070 (0.068, 0.073) | 1.44 (1.36, 1.52) | 0.008 (0.005, 0.011) | 0.052 (0.050, 0.055) | 1.18 (1.11, 1.25) |
| Living with others | 0.021 (0.015, 0.027) | 0.070 (0.064, 0.075) | 1.42 (1.30, 1.55) | 0.008 (0.001, 0.015) | 0.052 (0.045, 0.059) | 1.17 (1.02, 1.33) |
| Other | 0.019 (0.011, 0.026) | 0.068 (0.061, 0.075) | 1.38 (1.23, 1.53) | 0.031 (0.017, 0.044) | 0.075 (0.062, 0.088) | 1.69 (1.39, 1.99) |
| **60-69 years** | N=373,426 |  |  | N=447,311 |  |  |
| Living with a partner only | Ref | 0.093 (0.092, 0.095) | Ref | Ref | 0.075 (0.073, 0.077) | Ref |
| Living with a partner & 1+ minor child | 0.004 (-0.004, 0.013) | 0.098 (0.089, 0.106) | 1.04 (0.95, 1.14) | 0.000 (-0.028, 0.028) | 0.075 (0.047, 0.103) | 1.00 (0.63, 1.37) |
| Living with a partner & adult children | 0.006 (0.002, 0.010) | 0.100 (0.096, 0.104) | 1.07 (1.02, 1.11) | 0.007 (0.003, 0.011) | 0.082 (0.078, 0.086) | 1.09 (1.03, 1.14) |
| Lone parent living with 1+ minor child | 0.018 (-0.032, 0.067) | 0.111 (0.061, 0.161) | 1.19 (0.66, 1.72) | -0.003 (-0.061, 0.056) | 0.072 (0.014, 0.131) | 0.97 (0.19, 1.75) |
| Lone parent living with adult children | 0.014 (-0.001, 0.029) | 0.107 (0.093, 0.122) | 1.15 (0.99, 1.31) | 0.006 (0.000, 0.013) | 0.081 (0.076, 0.087) | 1.08 (0.99, 1.17) |
| Living alone | 0.029 (0.023, 0.035) | 0.122 (0.118, 0.127) | 1.31 (1.24, 1.38) | 0.007 (0.003, 0.012) | 0.083 (0.080, 0.085) | 1.10 (1.04, 1.16) |
| Living with others | 0.023 (0.014, 0.032) | 0.116 (0.108, 0.125) | 1.24 (1.14, 1.35) | 0.015 (0.008, 0.023) | 0.090 (0.084, 0.097) | 1.20 (1.10, 1.31) |
| Other | 0.102 (0.079, 0.124) | 0.195 (0.172, 0.217) | 2.09 (1.84, 2.34) | 0.155 (0.124, 0.186) | 0.230 (0.199, 0.261) | 3.07 (2.64, 3.49) |
| **70-79 years** | N=221,558 |  |  | N=361,794 |  |  |
| Living with a partner only | Ref | 0.195 (0.191, 0.199) | Ref | Ref | 0.165 (0.157, 0.173) | Ref |
| Living with a partner & adult children | 0.016 (0.006, 0.025) | 0.210 (0.201, 0.220) | 1.08 (1.03, 1.13) | 0.012 (0.002, 0.023) | 0.177 (0.165, 0.190) | 1.08 (1.01, 1.14) |
| Lone parent living with adult children | 0.013 (-0.009, 0.036) | 0.208 (0.187, 0.230) | 1.07 (0.95, 1.19) | 0.016 (0.002, 0.030) | 0.181 (0.172, 0.189) | 1.10 (1.01, 1.19) |
| Living alone | 0.018 (0.005, 0.031) | 0.213 (0.203, 0.223) | 1.09 (1.02, 1.16) | 0.011 (-0.001, 0.023) | 0.176 (0.171, 0.181) | 1.07 (0.99, 1.14) |
| Living with others | 0.003 (-0.015, 0.021) | 0.198 (0.181, 0.214) | 1.01 (0.92, 1.11) | 0.033 (0.018, 0.047) | 0.197 (0.189, 0.206) | 1.20 (1.10, 1.29) |
| Other | 0193 (0.160, 0.226) | 0.387 (0.355, 0.420) | 1.99 (1.81, 2.17) | 0.227 (0.204, 0.251) | 0.392 (0.371, 0.413) | 2.38 (2.20, 2.56) |
| **80-89 years** | N=65,474 |  |  | N=162,295 |  |  |
| Living with a partner only | Ref | 0.330 (0.318, 0.343) | Ref | Ref | 0.331 (0.308, 0.355) | Ref |
| Living with a partner & adult children | -0.020 (-0.043, 0.003) | 0.310 (0.286, 0.335) | 0.94 (0.87, 1.01) | -0.027 (-0.060, 0.005) | 0.304 (0.266, 0.342) | 0.92 (0.82, 1.01) |
| Lone parent living with adult children | 0.025 (-0.015, 0.065) | 0.355 (0.321, 0.389) | 1.08 (0.95, 1.20) | -0.023 (-0.052, 0.006) | 0.309 (0.296, 0.321) | 0.93 (0.85, 1.01) |
| Living alone | 0.026 (0.000, 0.053) | 0.357 (0.340, 0.373) | 1.08 (0.99, 1.16) | -0.009 (-0.035, 0.018) | 0.323 (0.318, 0.328) | 0.97 (0.90, 1.05) |
| Living with others | 0.052 (0.019, 0.085) | 0.382 (0.356, 0.408) | 1.16 (1.05, 1.26) | 0.015 (-0.014, 0.043) | 0.346 (0.336, 0.357) | 1.04 (0.96, 1.13) |
| Other | 0.067 (0.032, 0.103) | 0.398 (0.367, 0.428) | 1.20 (1.09, 1.32) | 0.061 (0.032, 0.091) | 0.393 (0.378, 0.407) | 1.19 (1.09, 1.29) |

Ref: reference category

Adjusting for current age dummies, region of residence, education, household income, labour force status, and marital status at time of entry to the age group
